# Sequencing and Bioinformatics-Based Analyses of the microRNA Transcriptome in Hepatitis B–Related Hepatocellular Carcinoma

**DOI:** 10.1371/journal.pone.0015304

**Published:** 2011-01-25

**Authors:** Yoshiaki Mizuguchi, Takuya Mishima, Shigeki Yokomuro, Yasuo Arima, Yutaka Kawahigashi, Kengo Shigehara, Tomohiro Kanda, Hiroshi Yoshida, Eiji Uchida, Takashi Tajiri, Toshihiro Takizawa

**Affiliations:** 1 Department of Surgery for Organ Function and Biological Regulation, Nippon Medical School, Tokyo, Japan; 2 Department of Molecular Anatomy and Medicine, Nippon Medical School, Tokyo, Japan; The University of Hong Kong, China

## Abstract

MicroRNAs (miRNAs) participate in crucial biological processes, and it is now evident that miRNA alterations are involved in the progression of human cancers. Recent studies on miRNA profiling performed with cloning suggest that sequencing is useful for the detection of novel miRNAs, modifications, and precise compositions and that miRNA expression levels calculated by clone count are reproducible. Here we focus on sequencing of miRNA to obtain a comprehensive profile and characterization of these transcriptomes as they relate to human liver. Sequencing using 454 sequencing and conventional cloning from 22 pair of HCC and adjacent normal liver (ANL) and 3 HCC cell lines identified reliable reads of more than 314000 miRNAs from HCC and more than 268000 from ANL for registered human miRNAs. Computational bioinformatics identified 7 novel miRNAs with high conservation, 15 novel opposite miRNAs, and 3 novel antisense miRNAs. Moreover sequencing can detect miRNA modifications including adenosine-to-inosine editing in miR-376 families. Expression profiling using clone count analysis was used to identify miRNAs that are expressed aberrantly in liver cancer including miR-122, miR-21, and miR-34a. Furthermore, sequencing-based miRNA clustering, but not individual miRNA, detects high risk patients who have high potentials for early tumor recurrence after liver surgery (*P* = 0.006), and which is the only significant variable among pathological and clinical and variables (*P* = 0,022). We believe that the combination of sequencing and bioinformatics will accelerate the discovery of novel miRNAs and biomarkers involved in human liver cancer.

## Introduction

Hepatocellular carcinoma (HCC) ranks in prevalence and mortality among the top 10 cancers worldwide [**1]**, [2], [3****]. The estimated number of new cases of HCC had risen to 564 300 and 548 600 people with HCC had died, representing 97.2% of persons with this diagnosis. The WHO estimates that 2 billion people worldwide have been infected with the hepatitis B virus (HBV), which is the most common cause of HCC, and that 350 million people have chronic HBV infection. As the current therapeutic options for HCC patients are limited, there is an urgent need to analyze the molecular oncogenic mechanisms in order to determine novel targets for specific systemic therapy and to detect novel biomarkers for early diagnosis.

MicroRNAs (miRNAs) participate in crucial biological processes, including development, differentiation, apoptosis, and proliferation [**4]**, [5****] through imperfect pairing with target messenger RNAs (mRNAs) of protein-coding genes and transcriptional or post-transcriptional regulation of their expression [**6]**, [7****]. Approaches to miRNA detection, such as parallel sequencing technologies may replace conventional sequencing[**8**]. The GS 454 technology can produce a similar number of longer (100–150-nucleotides (nt)) sequence reads in a single analysis run[**9**], with the advantage that this method can derive the complete sequence of the mature miRNA. Moreover, recent studies on miRNA profiling performed with cloning techniques suggest that sequencing methods are suitable for the detection of novel miRNAs, modifications, and precise compositions, and that cloning frequencies calculated by clone count analysis strongly correlate with the concentrations measured by Northern blotting, and are reproducible. The achievement of comprehensive profiling of miRNA in human diseases requires exhaustive qualitative and quantitative analyses. Here we focused on the sequencing method to profile and characterize miRNA transcriptomes in the liver. This serves as a critical step in clarifying the functional significance of specific miRNAs as they relate to HCC.

## Materials and Methods

### Study populations, liver tissues, and cell lines

The study material comprised 22 pairs of HBV-associated HCC liver samples obtained from patients undergoing liver surgery at the Department of Liver Surgery, Nippon Medical School Hospital (**Table S1**). The study protocol was approved by the Human Ethics Committee of the Nippon Medical School and the Research Ethics Committee of the Nippon Medical School. Patients who signed on the written informed consent form were enrolled in this study. All of the patients were positive for circulating hepatitis B surface antigen (HBsAg) or antibody to hepatitis B core antigen (anti-HBc) and were negative for antibody to hepatitis C virus (anti-HCV), autoimmune liver disease, primary biliary cirrhosis, primary sclerosing cholangitis, and concomitant inflammatory connective tissue disorder. None of the patients had past or present medical histories of alcohol abuse or long-term consumption of corticosteroids or tamoxifen. Normal liver tissues adjacent to the cancer were used as controls. In addition, six normal liver samples devoid of any cancer were included in the study. The three human HCC cell lines, JHH-7, HuH-1, PLC/PRF/5, Hep3B which were infected with HBV, were obtained from the Cancer Cell Repository at Tohoku University, Sendai, Japan. All of the cell lines were maintained for less than 6 months from when the suppliers sent them and were handled as recommended by the suppliers at 37°C and 5% CO_2_ in a humidified incubator.

### Conventional cloning and 454 sequencing of miRNAs

**Figure 1:**
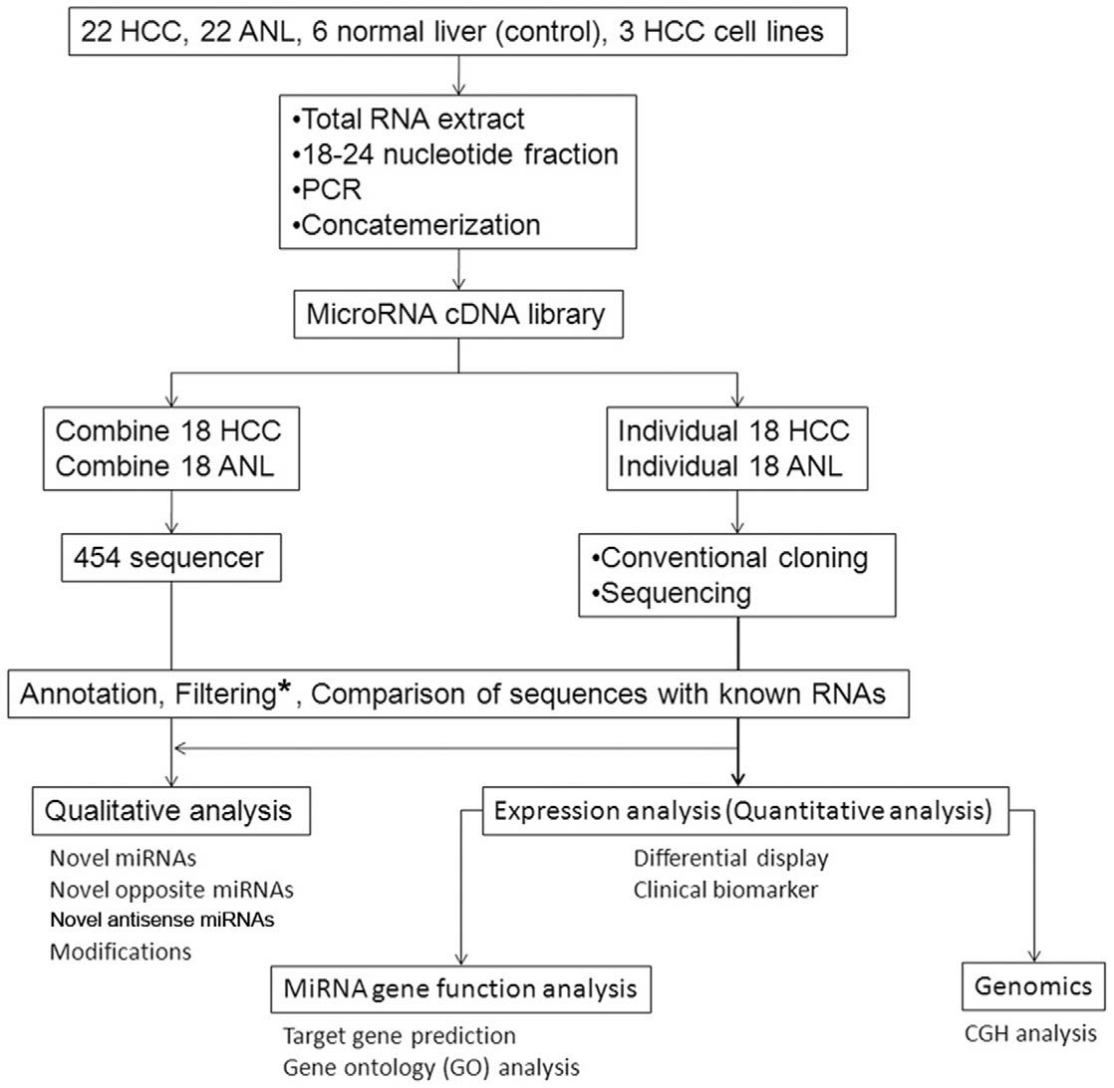
Overview algorithm of the miRNA analysis in this study. ^*^ Filtering indicates filtering of t/r/sn/sno/m/piRNAs. HCC, hepatocellular carcinoma; ANL, adjacent normal liver; cDNA, complementary DNA; CGH, comparative genomic hybridization.

We cloned small RNA by a modification of the published miRNA cloning protocol[**10**]. In brief, total RNA samples were extracted using ISOGEN (Nippon Gene, Tokyo, Japan), separated in a denaturing polyacrylamide gel, and the 18–24 nt fraction was recovered. Next, 5′- and 3′-adapters were ligated to the RNAs and RT-PCR was carried out. Amplification of the cDNA fragments was obtained by two consecutive rounds of PCR. Specific restriction enzyme digestion of the adaptors allowed for concatemerization of the cDNA into larger fragments. These fragments were then cloned into a vector to create a cDNA library. Concatemerization increases the length of informative sequences obtainable from each clone. The sequences were compared to human DNA to determine the genomic origin of the small RNA. For expression analysis, the RNA sequences from the clones were annotated and normalized per 1 000 reads. For 454 analysis, combined cDNA from HCC samples positive for hepatitis surface antigen (n = 18) was compared to combined cDNA from adjacent normal liver (ANL) samples (n = 18) (**Figure 1**).

### Bioinformatics analysis of the sequence data

#### Comparing the cloned sequences with those of known RNAs

The small RNA sequences were analyzed for homology with known RNAs, including miRNA, piwi-interacting (pi) RNA, rRNA, tRNA, small nuclear (sn) RNA, small nucleolar (sno) RNA, and mRNA, and human genomic DNA sequences. The databases used were: miRNA (mature- and pre-), Sanger Database.; piRNA, the NCBI Entrez Nucleotide database; rRNA, the European ribosomal RNA database; tRNA, the Genomic tRNA database; sn/snoRNA, RNAdb and NONCODE; mRNA, NCBI Reference Sequence; and human genomic sequences, the UCSC Genome Bioinformatics Site. In our searches, we defined the cloned sequencing results that had higher than 90% homology as valid if they met our criteria for sequence error, erroneous PCR amplification, and 3′- and 5′-end variations. Clones that shared 100% homology with human genomic DNA but did not match known RNAs when compared to the above databases were termed novel miRNA candidates and were subjected to further analysis.

#### Secondary structure analysis

The two-dimensional precursor miRNA (pre-miRNA) configurations of our novel miRNA candidates were predicted according to the method described previously [**11**] with some modifications. Briefly, 196-nt of genomic sequence was added to the candidate sequences (88-nt at each end). Each candidate sequence was divided into 110-nt windows and subjected to two-dimensional analysis along its entire length, using the RNAfold software (Vienna RNA Secondary Structure Package[**12**]). The configurations that had the lowest free energy and that had a high conservation (described below) and met the following criteria were termed novel miRNAs: (*a*) contained a stem-loop configuration; (*b*) cloned mature miRNA sequence portion consisted of more than 16-nt in its double-stranded region; (*c*) the loop contained fewer than 20-nt; (*d*) the internal loop contained fewer than 10-nt; and (*e*) the bulge contained fewer than 5-nt. Furthermore, novel sequences with overlapping positions in the genome were grouped together. Novel antisense miRNAs are defined with above criteria (a)-(e) but without conservation score if they are coded in same chromosomal region.

#### Determination of hairpin conservation

We classified all the candidate miRNAs using the PhastCon database at the University of California at Santa Cruz[**13]**, [14****]. This database has scores for each nucleotide in the human genome relative to its degree of conservation when compared to nucleotides in the armadillo, bush baby, cat, chicken, chimpanzee, cow, dog, elephant, frog, fugu, guinea pig, hedgehog, horse, lizard, medaka, mouse, opossum, platypus, rabbit, rat, rhesus monkey, shrew, stickleback, tenrec, tetraodon, tree shrew, and zebrafish. The algorithm is based on a phylogenetic hidden Markov model that uses best-in-genome pairwise alignment for each species (based on BLASTZ), followed by multiple alignment of the twenty eight genomes. A hairpin was defined as conserved if the average PhastCon conservation score over the 28 species for any 15-nt sequence in the hairpin stem was at least 0.8[**15**].

#### Hierarchical clustering of miRNA expression

All the 53 samples were analyzed - 22 HCC serologically positive for HBsAg, 22 ANL, 3 HBsAg positive cancer cell lines, and 6 normal liver tissues clustered using miRNA cloning. Clustering analysis was performed with cloned mature miRNAs. Mature miRNAs that were expressed in fewer than 25% of the samples were not subjected to analysis. The samples are listed in the columns, and the miRNAs are listed in the rows.

#### Gene ontology analysis

The predicted targets of hsa-miR-21, hsa-miR-34a, and hsa-miR-122 were determined using the miRBase Targets Beta version 1.0 and Target Scan programs and analyzed with respect to overrepresentation within different biological grouping categories, including gene ontology (GO). The level of overrepresentation was measured using Fisher's exact test in a 2×2 contingency table for each GO term (whether a gene is in the given list or not versus whether this gene is associated with a GO term or not). The Fisher's exact test *P* values were calculated for each term in each GO and ranked from smallest to highest values, to estimate the statistical significance and priority for each term.

#### Detection and determination of RNA modification sites

Two independent measurements (conventional cloning method and 454 sequencing) were performed, generating identical values. For miR-376c, miR-376a, miR-34a, miR-503, miR-21, and miR-122, the RNA modifications were detected using both methods, and the possibility of a single nucleotide polymorphism at any of the RNA editing sites was excluded by comparison with the public database OncoDb HCC. Adenosine (*A*)-to-inosine (*I*) editing was identified as a novel guanosine (*G*) peak and a drop in peak height at the *A*s in a cDNA sequence relative to the equivalent peak in the matching genomic DNA sequence. *X*-to-*Y* editing was identified as a novel *Y* peak and a drop in peak height at the *X*s. The drop in peak height between the DNA reference trace and the cDNA trace at the modified nucleotide is reported as a percentage of the peak height in the genomic DNA reference trace. For each modified miRNA, the mean extent of modification for each tissue is calculated from all the cDNA sequences obtained from that tissue.

#### PCR analysis of novel miRNAs

After bioinformatic analysis of the sequence data, we further validated novel miRNAs by PCR-based miRNA detection [**16**]. Briefly, small RNAs from Hep3B cell line were isolated using the mirVana™ miRNA isolation kit (Ambion). Small RNA samples were polyadenylated with Poly (A) Tailing Kit (Ambion) and were purified with Acid-Phenol:Chloroform and with filter cartridge provided in the mirVana Probe & Marker Kit (Ambion). To generate a small RNA cDNA library, tailed RNA were reverse transcripted using RTQ primer [**16**] and the samples were purified using the QIAquick spin PCR purification kit (QIAGEN). A small RNA-specific primer and a universal reverse primer (**Table S2**), RTQ-UNIr [**16**], were used for amplification of each of the small RNAs. The PCR products were analyzed on a 12% polyacrylamide gel. The primers for the human GAPDH were used for negative control.

### Clinicopathologic variables

The clinicopathologic variables were selected according to the general rules for the clinical and pathologic study of primary liver cancers[**17**], i.e., TNM stage[**18**], histological differentiation, growth type, capsule formation (fc), capsule invasion (fc-inf), septum formation (sf), serosa invasion (s), portal vein invasion (vp), venous invasion (vv), arterial invasion (a), bile duct invasion (b), and intrahepatic metastasis (im). Other factors studied included the patient's age, gender, and hepatitis virus.

### Statistical methods

A *P* value of <0.05 was considered statistically significant, and all tests were two-tailed. All interval values are expressed as mean ± SD. Statistically significant differences between two groups were calculated by the paired Student *t* test. For categorical comparisons of the data, the χ^2^ test or Fisher's exact test was used. To detect miRNAs with significant differences between HCC and ANL, we used SAM, which is a statistical technique for finding significant genes originally in a set of microarray experiments.[**19**] SAM computes a statistic *di* for each gene *i*, measuring the strength of the relationship between miRNA gene expression and the response variable. It uses repeated permutations of the data to determine whether the expression of any specific gene is significantly related to the response. We determined the cut-off for significance using a tuning parameter delta, chosen on the basis of a false-positive rate of zero. The array data was used to construct receiver-operating-characteristic curves by plotting the relationships of the true positives (sensitivity) and false positives (1-specificity). For survival analysis, we computed the statistical significance for each variable based on a Cox proportional hazards regression model. The Kaplan-Meier method was used to estimate the patient survival and recurrence curve, and the log-rank test was used to compare the subgroups. A multiple Cox proportional hazards regression model was used to investigate the multiple variables individually for a possible association with survival. We introduced all of the pathologic variables and age and gender (**Table S1**) into the Cox regression model.

### Accession Numbers

The reported novel miRNAs we have deposited with DDBJ under accession codes as indicated.

### Websites for database used in this manuscript

NCBI Entrez Nucleotide database, http://www.ncbi.nlm.nih.gov/entrez/query.fcgi?db=Nucleotide; European ribosomal RNA database, http://psb.ugent.be/rRNA/; Genomic tRNA database, http://lowelab.ucsc.edu/GtRNAdb/; RNAdb, http://research.imb.uq.edu.au/randb/; NONCODE, http://www.bioinfo.org.cn/NONCODE/; NCBI Reference Sequence, ftp://ftp.ncbi.nih.gov/refseq/; UCSC Genome Bioinformatics Site, http://genome.ucsc.edu; OncoDb HCC, http://oncodb.hcc.ibms.sinica.edu.tw/index.

## Results

### Qualitative analysis of miRNAs using sequencing and prediction of novel and various coding of precursor miRNAs in human liver

To obtain the miRNA gene expression profiles of liver tissues from patients with HBV associated HCC (**Table S1**), we combined conventional cloning with GS 454 sequencing technology (**Figure 1**). We introduced concatemerization to increase the length of the informative sequence obtained from each sequence read. Software programs and databases were used for the identification and annotation of miRNAs. We identified more than 314 000 reliable reads from HCC and more than 268 000 from ANL for human miRNAs registered in miRBase (**Table S3**). A high percentage (approximately 80%) of the clone reads (>400 000) was matched to annotated human miRNA sequences (**Table S4**). A low percentage of the clone reads matched annotated m/t/r/sn/snoRNA. Unexpectedly, more than 4 400 (0.76%) reads were annotated as piRNA.

**Figure 2:**
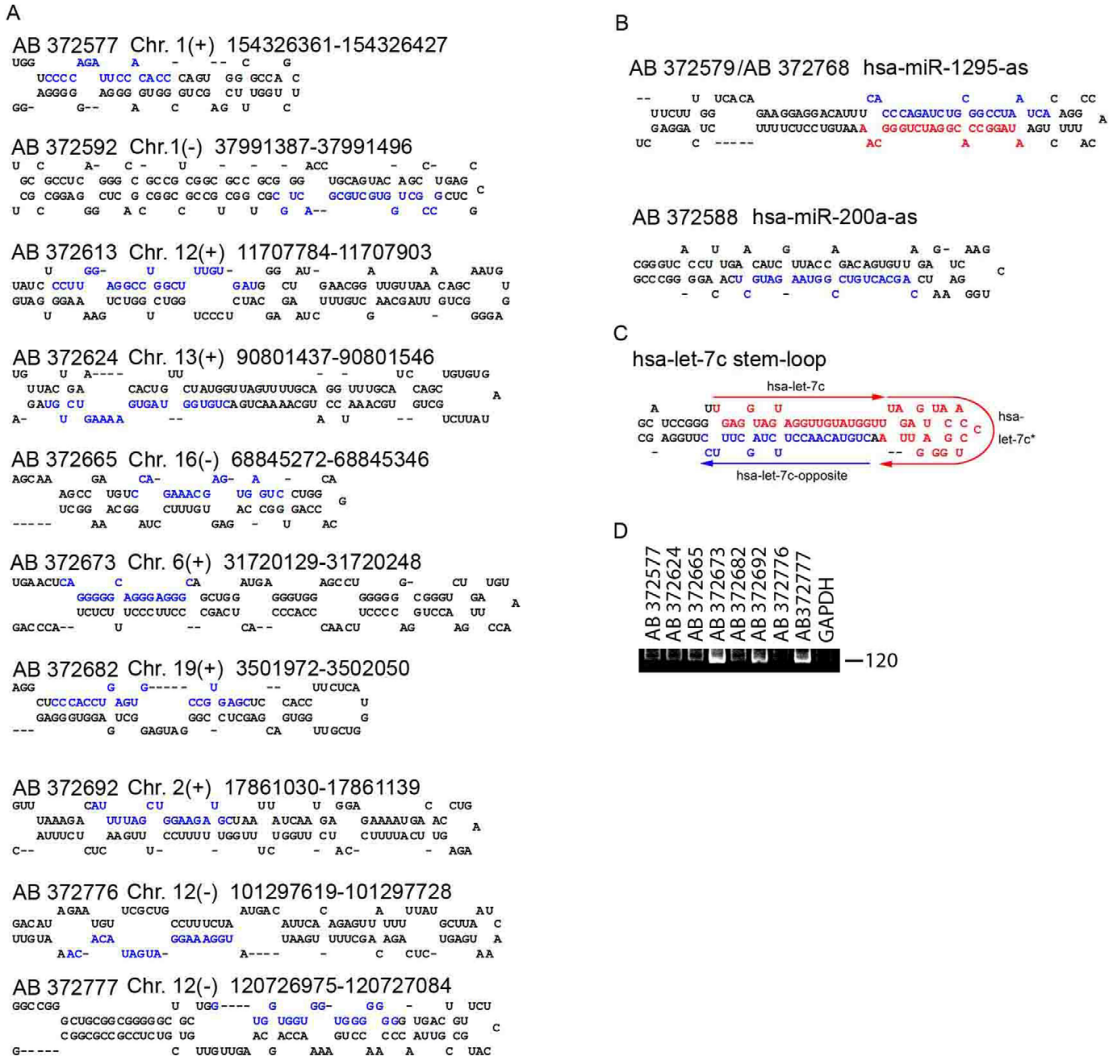
Qualitative analysis of miRNAs in the human liver. (**A**)**–**(**C**) Secondary structure and genome location of novel miRNAs (**A**), novel antisense miRNA (**B**) and pre- let7C (**C**). The cloned novel and known mature miRNA sequences are indicated in blue or red, respectively. (**D**) Analysis of novel miRNAs using semiquantitative PCR. A representative gel picture shows the expression of novel miRNAs. GAPDH was used as a negative control. The numbers following AB (e.g., AB 372796) are accession codes we have been depositing with DDBJ. (See Materials and Methods).

Based on these criteria, a scan of all the novel miRNA candidates (>250) identified 10 novel precursors with evolutionarily high conservation, representing putative 10 novel mature miRNAs (**Figure 2A** **and Table S5**). Fifteen novel opposite miRNAs (defined as miRNAs cloned from the other arm of precursors from which known miRNAs have been cloned) were identified from the annotated miRNAs (**Table S6**). And also three novel antisense miRNAs were identified from the annotated miRNAs (**Figure 2B** **and **Table S5****). Although opposite miRNAs are inferred to exist for all miRNAs, some miRNAs (e.g., hsa-miR-503) with many clone reads have no opposite sequence. Unexpectedly, bioinformatics analysis and secondary structure predictions of the novel miRNA candidates allow the 7c precursor to produce three non-overlapping mature miRNAs from the 5′-pole, 3′-pole, and loop, respectively (**Figure 2C**). In the present study, the highly conserved miRNAs of the let-7 family exhibited opposite miRNAs, with the exception of let-7a-2, and the sequences were highly similar to each other, generating six distinct seeds (i.e., nt 1 or nt 2–8 of the mature miRNA) (**Figure S1**), thereby supporting our results. We then probed to confirm whether these 10 candidates for novel miRNA could be detected using PCR-based small RNA detection method. Seven of 10 candidates were specifically detected by the method, which indicates that these candidates were indeed novel miRNAs (**Figure 2D**). Specific bands for the other candidates like AB372776 and band for the GAPDH were undetectable (**Figure 2D**).

### Detection of various RNA modifications in mature miRNAs

**Figure 3:**
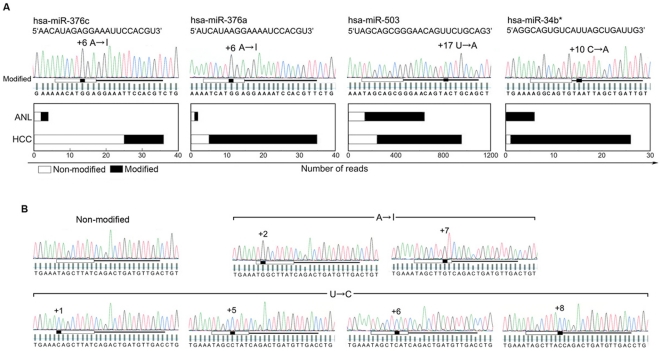
RNA modifications of mature miRNAs in the human liver. (**A**) Sequence analysis with chromatogram of mature miRNAs for which the modified forms are predominantly expressed in the liver. Modified miR-376c, miR-376a, miR-34b*, and miR-503 are detected by RT-PCR and conventional cloning. An *A*→*I* modification is shown as *A*→*G*, and *U*→*A* editing is shown as *T*→*A*. The horizontal bars under the chromatograms indicate the miRNA site with the 5′-proximal seed region marked by a thick white bar. Nucleotide substitutions are highlighted in black. The modified sites are numbered with the 5′-end of the mature miRNA sequence designated as +1. The vertical bars located below each peak of the chromatogram are quality values bars which indicate the veracity of each base call. A horizontal line is placed across the quality bars. The bars above this line indicate base calls that have a more than 99% probability of being correct. The bars underneath this line have a less than 99% probability of being correct. The frequency of modified and non-modified clone reads for HCC and ANL from the 454 sequencer are charted in the bar graphs below the chromatograms. (**B**) Sequence analysis with chromatogram of mature miR-21, in which *A*→*I* or *U*→*C* modification occurs in the 5′-proximal seed sequence. The miRNA site, 5′-seed region, and base call quality value bars are as described above.

A recent study has reported tissue-specific editing of miR-376 cluster members and the expression of edited mature miR-376 RNAs in certain tissues [**20**]. Next, we focused on the sequence compositions of cloned mature miRNAs. In general, these analyses indicate that for a given mature miRNA there are many sequence compositions. The 5′-proximal end sequence (+1) was almost always conserved. In contrast, the 3′-end sequence (1–3 nt) showed many variations, presumably resulting from incomplete participation of RNase III in the process of biogenesis. Thus, in principle, the seed sequences are highly conserved in these clones (>98%). Scanning of the sequence compositions revealed that certain miRNAs were predominantly substituted at specific positions, even at the seed positions (**Figure 3A**). First, in miR-376c and miR-376a, members of miR-376 cluster, specific adenosine (*A*) residues in the seed sequence (+6 site) were detected as guanosine (*G*) in the sequencing chromatogram, which suggests adenosine to inosine (*I*) editing [**20**]. Overall, 31% and 50% of the miR-376c and 86% and 50% of the miR-376a were expressed as the edited form in HCC and ANL, respectively. Second, a component of the p53 tumor suppressor network, miR-34b*, belongs to an evolutionarily conserved miR-34 family (miR-34a-c), with single, recognizable orthologues in several invertebrate species[**21**]. The miR-34b* is not conserved at the +10 position between humans and rodents. Of particular interest, almost all the miR34b* forms are expressed with *C*→*A* editing at the +10 position (HCC, 96%; ANL, 100%) in the liver, resulting in the same sequence as those observed in the mouse and rat. In contrast, no modifications were detected in miR-34a and miR-34c. Third, 74% and 78% of miR-503 expressed *U*→*A* at the +17 position in the modified forms in HCC and ANL, respectively.

Further investigation of the mature miRNAs at positions +1 to +18 (excluding the 3′*-*variable sequences) revealed that, although not predominant (∼5%), modified sequences were present in almost all the miRNAs. In the analysis of the top 50 (**Table S7**) sequences of the mature miR-122 and miR-21, *A*→*I* modifications, *U*→*C*, *G*→*A*, *U*→*A*, and *C*→*U* modifications were observed, as evidenced by the presence of a single peak (**Figure 3B**). *U*→*C* editing has been suggested only for primary miRNA (pri-miRNAs) [**22**], and other modification types have not been reported for pri-mRNAs and mature miRNAs. In the mature miR-21, modified forms for each *A*→*I* (+2 and +7) and *U*→*C* (+1, +5, +6, +8) at the seed sequence were detected, whereas in the mature miR-122, *A*→*I* editing was not detected (**Figure 3B**, **Figure S2**). These results strongly suggest that these modifications should be considered as confounding factors for accurate and precise miRNA expression profiles using hybridization-based methods.

### Detection of aberrant expressing miRNAs in human liver cancers using clone count analysis

**Figure 4:**
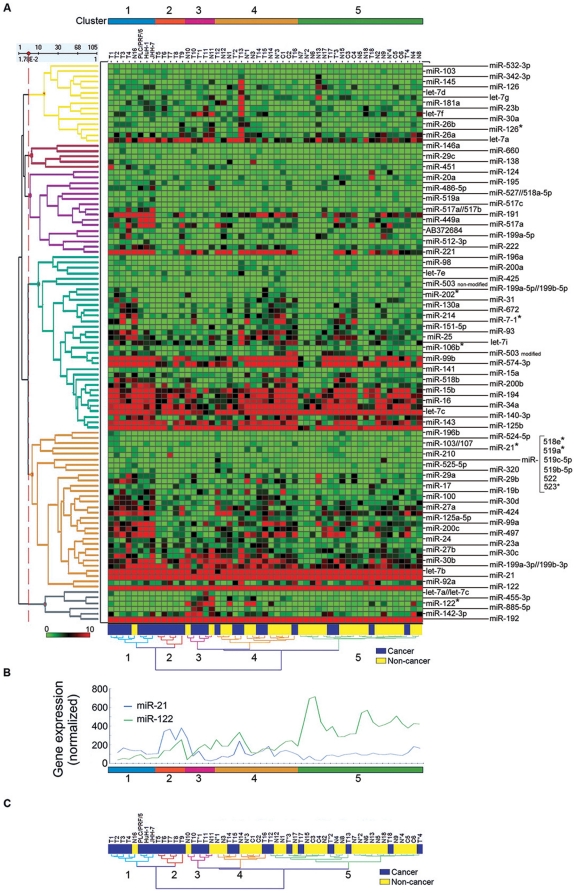
Quantitative expression profiles of miRNA in the liver. (**A**) Clustering of the miRNA profiles of 53 liver samples. Clustering analysis was performed with cloned mature miRNAs. Mature miRNAs that were expressed in fewer than 25% of the samples were not subjected to analysis. The samples are listed in columns, and the miRNAs are listed in rows. Samples of HCC origin are shown in blue and ANL in yellow at the bottom of the array. The HCC-derived samples generally cluster together in clusters 1 and 2. A sample number with an asterisk indicates a case of historical infection with HBV. (**B**) Expression levels of miR-21 (blue) and miR-122 (green) in the liver. (**C**) Example of how the clustering profile changes when one does not consider modified forms. Here is a clustering analysis when one does not consider modified miR-503.

**Figure 5:**
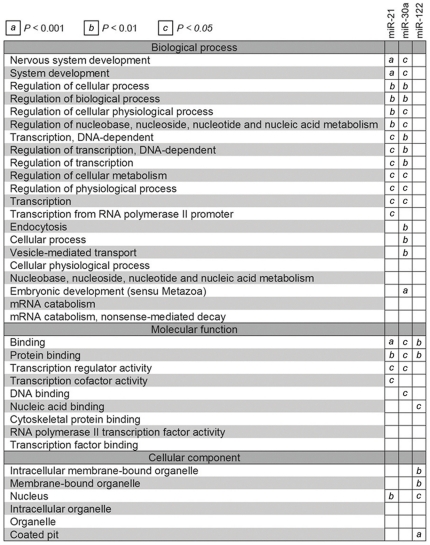
Gene ontology (GO) term comparison of aberrantly expressed miRNA targets. The predicted gene targets for aberrantly expressed miRNAs (miR-21, miR-34a, and miR-122) were compared to GO terms under the categories of ‘biological process,’ ‘molecular function,’ and ‘cellular component.’ The figure shows those terms that are overrepresented in the predicted gene targets of the miRNAs compared with all the Ensembl genes. All values are classified by their significance; the cells marked “*a*” (*P*<0.001) indicate that a miRNA specifically targets genes in that GO functional class.

It has become evident that miRNA alterations are involved in the initiation and progression of human cancer[**23]**, [24], [25****]. Using sequence-based methods, we set out to determine the expression patterns of miRNAs, including novel miRNAs, across a panel of samples representing human HBV associated HCC. The samples from malignant and benign liver specimen were clustered together using miRNA cloning, which confirms the ability to distinguish HCC from ANL (**Figure 4A**). The cancer cell lines showed similar expression profiles and were placed in cluster 1. Cluster 2 contained all the HCC samples with the distinct characteristics of high miR-21 (>250) expression (**Figure 4B**). When the clustering results were analyzed without taking into consideration the modified forms, the profiles were different from each other (**Figure 4C**). To evaluate statistically the differences in miRNA expression, 18 HCC samples serologically positive for HBsAg, the corresponding 18 ANL samples, and all the miRNAs listed in **Figure 4A** were introduced into student's-t test and SAM analysis (**Table S8**). In the student's-t analysis, seven miRNA genes in HCC had statistically higher expression levels and five had lower expression levels than those in ANL. Only the expression levels of the nonmodified form (not the modified form or the nonmodified plus modified form) of miR-503 were confirmed to be significantly different between HCC and ANL (*P* = 0.025). In the SAM analysis, two miRNAs were upregulated: (*a*) miR-21, which is overexpressed in many kinds of cancers and accelerates cell migration and invasion thorough phosphatase and tensin homolog (PTEN) in HCC [**26**]; and (*b*) miR-34a, which is a component of the p53 tumor suppressor network. Furthermore, miR-122 was downregulated, and it has been previously reported to be specifically expressed and highly abundant in human liver and required for the replication of HCV RNA[**27**]. To gain insight into the potential biological impact of aberrant miRNA expression in HCC, we identified Gene Ontology (GO) terms for the cellular process, molecular function, and cellular component categories[**28**], which were overrepresented in the predicted targets of aberrantly expressed miRNAs as compared with all the Ensembl genes (**Figure 5**). For the three aberrantly expressed miRNAs, analysis of overrepresented GO terms associated with the predicted targets of stronger- and weaker-expressing miRNAs revealed distinct differences.

### Potentials of miRNA sequencing and bioinformatics for detecting clinical biomarkers

**Figure 6:**
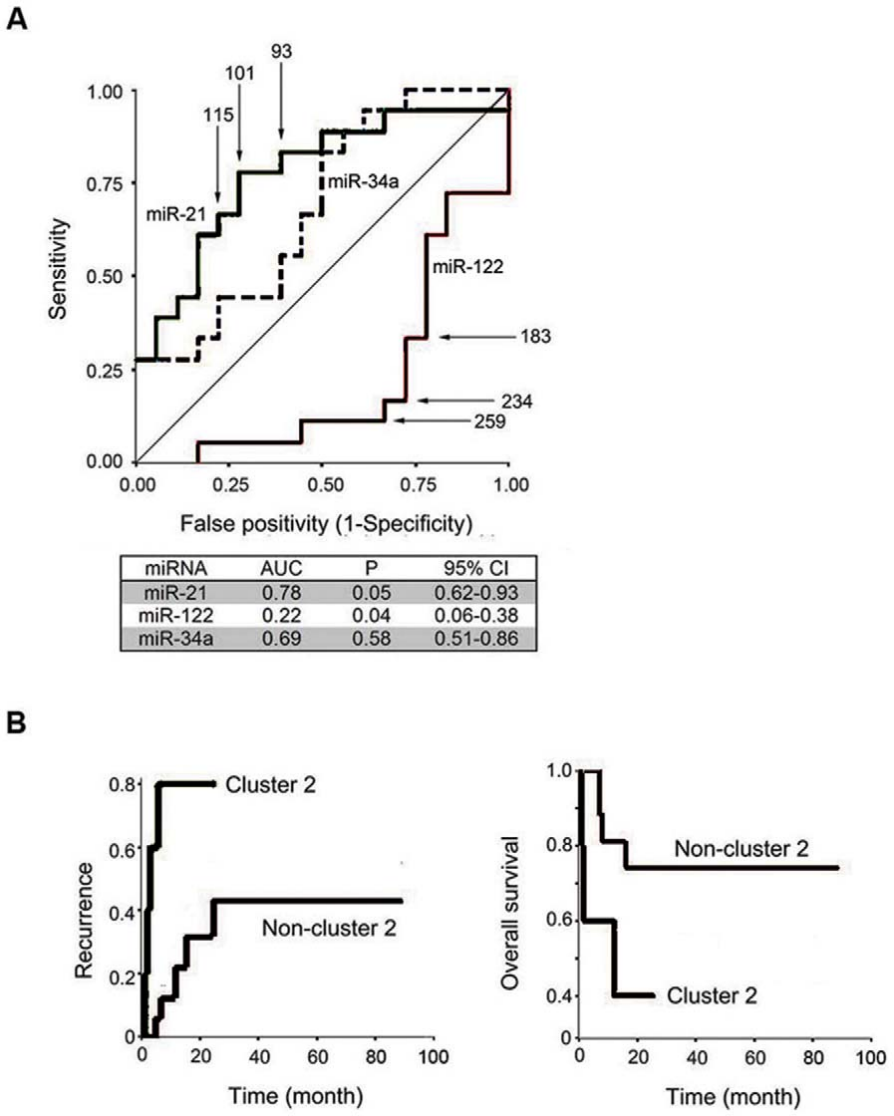
Potential application of sequencing-based miRNA clustering for the detection of clinical biomarkers. (**A**) Distinguishing HCC from adjacent normal liver using miRNA. This figure shows the receiver-operating-characteristic curve for using various cut-off points (indicated by arrows) of hsa-miR-21, has-miR-122 and has-miR34a. From the area under the curves, hsa-miR-21 and has-miR-122 are potential biomarkers for prediction of HCC. The true-positives (sensitivity) and false-positives (1-specificity), and the area under the receiver-operating-characteristic curve were comparatively analyzed. (**B**) Potential of sequencing-base miRNA clustering to detect predictive biomarkers for clinical course. The patients were divided into two groups according to the clustering analysis (cluster-2, n = 5; non-cluster-2, n = 17) (See Figure 4). The Kaplan-Meier method was used to determine the recurrence (left) and overall survival (right), and the log-rank test was used to compare the rates between the groups.

These data prompted the hypothesis that the miRNAs which are aberrantly expressed in HCC could be used as diagnostic biomarkers for HCC. As previously reported, the expression levels of miR-21 in HCC is significantly increased, whereas that of miR-122 is decreased. Thus we next studied whether expression levels of these miRNAs including miR-34a calculated with sequencing can also have potentials as clinical biomarkers. Receiver-operating-characteristic curves were constructed for the prediction of HCC (**Figure 6A**). From the analyses of the area under the curve, miR-21 and miR-122 appear to have potential as biomarkers for the prediction of HCC. The miR-21 cut-off point of 100 and the miR-122 cut-off point of 250 were the most reliable cut-off points in identifying ANL from HCC (**Table S9**). Having determined that the miRNAs expression levels distinguished tumors from non-tumors, we then considered whether miRNAs could be used as biomarkers for the clinical course of disease, particularly as predictors of survival and cancer recurrence after liver surgery. The individual miRNA Cluster 2 consisted exclusively of malignant tissues and had the prominent characteristic of miR-21 expression (>250), which was about 2-fold higher than the most reliable diagnostic cut-off point for HCC. Using the Kaplan-Meier test, and including the patients with historic HBV infection (n = 22), the patients in cluster 2 were found to have significantly (*P* = 0.0006) earlier recurrence time (mean, 7.6 months) than the other patients (mean, 57.6 mo) (**Figure 6B**, **Table S10**), whereas individual miR-21 or miR-122 could not predict patients' survival nor cancer recurrence after surgery (data not shown). In accordance with the cancer recurrence rate, although not statistically significant, the Kaplan-Meier analysis revealed that the patients in cluster 2 had a poorer survival rate than those in non-cluster 2 (mean, 13.4 vs. 69.6 month). Moreover, a Cox regression analysis performed on the pathology-oriented variables (**Table S11**) revealed that cluster 2 significantly and independently correlated with risk for poor prognosis, even after correction for confounding factors (*P* = 0.022). These results indicate that miRNA-21 has potential as a predictor of poor prognosis.

## Discussion

In this study, we demonstrate the usefulness and accuracy of sequencing in genetic research of the human liver cells by detecting minute alterations in the sequence leading to the discovery and identification of novel miRNAs, with differential manifestations between malignant and benign cases. One of the main problems with applying sequencing to the miRNA transcription research is that sequencing is a time-consuming procedure. And an important consideration for the discovery of miRNA by sequencing is the difficultly in identifying miRNAs that are expressed at low levels, at highly specific stages or in rare cell types. Moreover, a serious problem is that some miRNAs are difficult to profile precisely due to their physical properties or post-transcriptional modifications, such as RNA editing. [**29]**, [30****] In principle, these limitations can be overcome by extensive sequencing of small RNA libraries from a broad range of samples. For differential display, the sequencing-based method has the theoretical advantage in that it has the capability to discover and detect novel miRNAs. Based on our sequence variability results, especially with regard to RNA modifications, the accuracy of the sequence-based method is expected to be superior to that of the hybridization-based method. For the prediction of novel miRNAs, methods that rely on phylogenetic conservation of the structure and sequence of a miRNA cannot predict nonconserved genes. To overcome this problem, we made use of a computational approach for structural conservation criteria using the thermodynamic stability and intrinsic structural features of miRNAs. In clinics, pathologists often meet difficult situations in which they cannot clearly tell whether the tissue specimens they are observing are malignant or benign. Thus, in our opinion, using some miRNAs as a tumor marker would help clinicians to clearly determine whether that tissue is cancerous. As shown in Fig. 4, miRNA sequences followed by bioinformatics have greater power than individual miRNAs or other clinic-pathological variables for the detection of high risk patients' groups with poor prognoses. There is currently little data available as to how we can use each miRNA to predict high risk groups; however, additional future miRNA work and data accumulation will elucidate such criteria. And further investigation is warranted to clarify the mechanism of aberrant expression of miRNAs in cancer and its participation in carcinogenesis. Nevertheless, these findings show that sequence-based miRNA profiling has potential for the confirmation of precise miRNA dynamics in a specific disease. In addition, it will increase our understanding of the mechanisms and factors involved in human liver cancer.

## Supporting Information

Figure S1 **Multiple sequence alignments of cloned mature microRNAs of let7 family.** Multiple sequence alignment of cloned mature microRNAs from the let7 family derived from the 5′ stem (left column) and 3′ stem (right column) of the precursors. The alignments were generated with the ClustalW program. Conserved nucleotides of mature microRNAs were colored black (100%), dark gray (80–99%), and light gray (60–79%). let-7b*, most frequent sequence is not completely matched to genome (CUAUACAACCUACUGCCUUCCU); let-7e*, most frequent sequence is not completely matched to genome (CUAUACAGCCUCCUAGCUUUCCA); let-7c opposite sequence UAGAGUUACACCCUGGGAGUUA (underlined) is listed in miRBase (10.0), although this sequence is not detected in our study, whereas CUGUACAACCUUCUAGCUUUCC composition is detected.
(DOC)

Figure S2 **RNA modifications of mature miR-122 and miR-21in the human liver.** Sequence analyses of miR-122 (left column) and miR-21 (right column). The number of total (modified and nonmodified forms (**A**), modified forms (**B**), and individual modified sites (**C**) are indicated in the bar graph
(DOC)

Table S1 **Clinical and pathologic characteristics of the study population and cells used.**
(DOC)

Table S2 **Primers used for the PCR-based small microRNA detection.**
(DOC)

Table S3 **Summary of the cloned small RNAs.**
(DOC)

Table S4 **Summary of annotated miRNAs.** The clone counts from libraries of malignant and benign tissues and cell lines of the liver are summarized according to the type and subtype of miRNA. The miRNAs that have common cloned sequences are listed on a single line. The most frequent sequence is selected by the total number of clone counts, even if it does not match perfectly the genomic sequence. N, a sample from the adjacent normal liver; T, hepatocellular carcinoma; C, control; N* and T*, samples from the livers of patients having historic infection with HBV.
(XLSX)

Table S5 **Summary of predicted novel miRNAs.** The predicted novel miRNAs with their PhastCon scores are listed. The most frequent cloned sequences, genome locations, clone counts, and conservation scores calculated with PhastCon are listed. The precursor structures are listed in Fig. 1.
(DOC)

Table S6 **Summary of novel opposite miRNAs.** The novel opposite miRNAs with their precursor predicted structures, chromosomal locations, and number of clone reads are listed. The most frequent cloned sequence is listed, and the sequence that does not match the genomic sequence is shown in green. Comparison of the expression levels between miRNAs from the 3′-arm and 5′-arm are calculated with the paired Student's *t*-test.
(DOC)

Table S7 **Sequence compositions of mature has-miR21 and miR122.** Multiple sequence alignments were generated with the CLUSTALW program. Modified nucleotides at positions +1 to +18 are depicted in color [red, A→I (G); yellow, T→C; blue, T→A; green, C→T; pink, G→A] Nucleotides that have no modified sequences are colored black (100%), dark-gray (80–99%), and light-gray (60–79%). The gray colorations indicate the minus or plus position of the registered sequence. Note that there are particular modified patterns according to the particular nucleotide.
(DOC)

Table S8 **Results of the SAM and Student's *t*-test.**
(DOC)

Table S9 **Discriminating HCC from adjacent normal liver using miRNA.** The true-positives (sensitivity) and false-positives (1-specificity), together with the positive predictive values, negative predictive values, likelihood ratio for positive predictive value, negative predictive value, likelihood ratio for positive predictive value, and accuracy at various cut-off points are listed.
(DOC)

Table S10 **Analysis of the association between the postoperative clinical course and variables.** The postoperative recurrence and survival of patients with HCC in relation to pathologic tumor characteristics and miRNA expression calculated by clone counts are analyzed.
(DOC)

Table S11 **Cox regression analysis of the association between the postoperative clinical course and variables.**
(DOC)

## References

[1] Llovet JM, Burroughs A, Bruix J (2003). Hepatocellular carcinoma.. Lancet.

[2] Thorgeirsson SS, Grisham JW (2002). Molecular pathogenesis of human hepatocellular carcinoma.. Nat Genet.

[3] Lavanchy D (2004). Hepatitis B virus epidemiology, disease burden, treatment, and current and emerging prevention and control measures.. J Viral Hepat.

[4] Bartel DP (2004). MicroRNAs: genomics, biogenesis, mechanism, and function.. Cell.

[5] Harfe BD (2005). MicroRNAs in vertebrate development.. Curr Opin Genet Dev.

[6] Bartel DP, Chen CZ (2004). Micromanagers of gene expression: the potentially widespread influence of metazoan microRNAs.. Nat Rev Genet.

[7] Rajewsky N (2006). microRNA target predictions in animals.. Nat Genet.

[8] Meyers BC, Souret FF, Lu C, Green PJ (2006). Sweating the small stuff: microRNA discovery in plants.. Curr Opin Biotechnol.

[9] Margulies M, Egholm M, Altman WE, Attiya S, Bader JS (2005). Genome sequencing in microfabricated high-density picolitre reactors.. Nature.

[10] Lagos-Quintana M, Rauhut R, Yalcin A, Meyer J, Lendeckel W (2002). Identification of tissue-specific microRNAs from mouse.. Curr Biol.

[11] Mineno J, Okamoto S, Ando T, Sato M, Chono H (2006). The expression profile of microRNAs in mouse embryos.. Nucleic Acids Res.

[12] Hofacker IL (2003). Vienna RNA secondary structure server.. Nucleic Acids Res.

[13] Schwartz S, Kent WJ, Smit A, Zhang Z, Baertsch R (2003). Human-mouse alignments with BLASTZ.. Genome Res.

[14] Siepel A, Haussler D (2004). Combining phylogenetic and hidden Markov models in biosequence analysis.. J Comput Biol.

[15] Berezikov E, Guryev V, van de Belt J, Wienholds E, Plasterk RH (2005). Phylogenetic shadowing and computational identification of human microRNA genes.. Cell.

[16] Ro S, Park C, Jin J, Sanders KM, Yan W (2006). A PCR-based method for detection and quantification of small RNAs.. Biochem Biophys Res Commun.

[17] Japan LCSGo (2000). The General Rules for the Clinical and Pathological Study of Primary Liver Cancer..

[18] Sobin L, Wittekind C (2002). TNM Classification of Malignant Tumors..

[19] Tusher VG, Tibshirani R, Chu G (2001). Significance analysis of microarrays applied to the ionizing radiation response.. Proc Natl Acad Sci U S A.

[20] Kawahara Y, Zinshteyn B, Sethupathy P, Iizasa H, Hatzigeorgiou AG (2007). Redirection of silencing targets by adenosine-to-inosine editing of miRNAs.. Science.

[21] He L, He X, Lim LP, de Stanchina E, Xuan Z (2007). A microRNA component of the p53 tumour suppressor network.. Nature.

[22] Blow MJ, Grocock RJ, van Dongen S, Enright AJ, Dicks E (2006). RNA editing of human microRNAs.. Genome Biol.

[23] Calin GA, Dumitru CD, Shimizu M, Bichi R, Zupo S (2002). Frequent deletions and down-regulation of micro- RNA genes miR15 and miR16 at 13q14 in chronic lymphocytic leukemia.. Proc Natl Acad Sci U S A.

[24] Caldas C, Brenton JD (2005). Sizing up miRNAs as cancer genes.. Nat Med.

[25] Calin GA, Croce CM (2006). MicroRNA signatures in human cancers.. Nat Rev Cancer.

[26] Meng F, Henson R, Wehbe-Janek H, Ghoshal K, Jacob ST (2007). MicroRNA-21 regulates expression of the PTEN tumor suppressor gene in human hepatocellular cancer.. Gastroenterology.

[27] Jopling CL, Yi M, Lancaster AM, Lemon SM, Sarnow P (2005). Modulation of hepatitis C virus RNA abundance by a liver-specific MicroRNA.. Science.

[28] Ashburner M, Ball CA, Blake JA, Botstein D, Butler H (2000). Gene ontology: tool for the unification of biology. The Gene Ontology Consortium.. Nat Genet.

[29] Luciano DJ, Mirsky H, Vendetti NJ, Maas S (2004). RNA editing of a miRNA precursor.. RNA.

[30] Yang W, Chendrimada TP, Wang Q, Higuchi M, Seeburg PH (2006). Modulation of microRNA processing and expression through RNA editing by ADAR deaminases.. Nat Struct Mol Biol.

