# Supplementary material for: Sequencing and Bioinformatics-Based Analyses of the microRNA Transcriptome in Hepatitis B–Related Hepatocellular Carcinoma
Source: PLoS One. 2011 Jan 25;6(1):e15304. doi: 10.1371/journal.pone.0015304 (PMC3026781; doi:10.1371/journal.pone.0015304)
Supplement: Figure S1 — Multiple sequence alignments of cloned mature microRNAs of let7 family. Multiple sequence alignment of cloned mature microRNAs from the let7 family derived from the 5′ stem (left column) and 3′ stem (right column) of the precursors. The alignments were generated with the ClustalW program. Conserved nucleotides of mature microRNAs were colored black (100%), dark gray (80–99%), and light gray (60–79%). let-7b*, most frequent sequence is not completely matched to genome (CUAUACAACCUACUGCCUUCCU); let-7e*, most frequent sequence is not completely matched to genome (CUAUACAGCCUCCUAGCUUUCCA); let-7c opposite sequence UAGAGUUACACCCUGGGAGUUA (underlined) is listed in miRBase (10.0), although this sequence is not detected in our study, whereas CUGUACAACCUUCUAGCUUUCC composition is detected. (DOC) [file pone.0015304.s001.doc]

**Figure S1**

　　　　　 Known miRNA Opposite miRNA

-----ugggaUGAGGUAGUAGGUUGUAUAGUU-------uuagggucacacccaccacugggagauaaCUAUACAAUCUACUGUCUUUCCua----- :hsa-let7a-1

---ugugggaUGAGGUAGUAGAUUGUAUAGUU-------uuagggucauaccc-caucuuggagauaaCUAUACAGUCUACUGUCUUUcccacg--- :hsa-let7f-2

------aggcUGAGGUAGUAGUUUGUACAGUUugagggucuaugauaccaccc-gguacaggagauaaCUGUACAGGCCACUGCCUUGCca------ :hsa-let7g

-----cuggcUGAGGUAGUAGUUUGUGCUGUUggucggguugugacauugccc-gcugu-ggagauaaCUGCGCAAGCUACUGCCUUGCua------ :hsa-let7i

-----cggggUGAGGUAGUAGGUUGUGUGGUU-ucagggcagugauguugccc-c--ucggaagauaaCUAUACAACCUACUGCCUUCCcug----- :hsa-let7b

----ucagagUGAGGUAGUAGAUUGUAUAGUU-gugggguagugauuuuaccc-uguucaggagauaaCUAUACAAUCUAUUGCCUUCCcuga---- :hsa-let7f-1

-------gggUGAGGUAGUAGGUUGUAUAGUU--------uggggcucugccc-ug-cuaugggauaaCUAUACAAUCUACUGUCUUUCCu------ :hsa-let7a-3

gcauccggguUGAGGUAGUAGGUUGUAUGGUU-uagaguua---------cac---ccugggaguuaaCUGUACAACCUUCUAGCUUUCCuuggagc :hsa-let7c

---cccgggcUGAGGUAGGAGGUUGUAUAGUU-gaggagga---------cac---ccaaggagaucaCUAUACAGCCUCCUAGCUUUCCccagg-- :hsa-let7e

---ccuaggaAGAGGUAGUAGGUUGCAUAGUU-uuagggcagggauuuugccc---acaaggagguaaCUAUACGACCUGCUGCCUUUCUuagg--- :hsa-let7d

------agguUGAGGUAGUAGGUUGUAUAGUU-uagaauua---------cau---caagggagauaacuguacagccuccuagcuuuccu------ :hsa-let7a-2

**Figure S1.** Multiple sequence alignments of cloned mature microRNAs of let7 family. Multiple sequence alignment of cloned mature microRNAs from the let7 family derived from the 5’ stem (left column) and 3’ stem (right column) of the precursors. The alignments were generated with the ClustalW program. Conserved nucleotides of mature microRNAs were colored black (100%), dark gray (80-99%), and light gray (60-79%).

let-7b*,most frequent sequence is not completely matched to genome (CUAUACAACCUACUGCCUUCCU)

let-7e*, most frequent sequence is not completely matched to genome (CUAUACAGCCUCCUAGCUUUCCA)

let-7c opposite sequence UAGAGUUACACCCUGGGAGUUA (underlined) is listed in　miRBase(10.0), although this sequence is not detected in our study, whereas　CUGUACAACCUUCUAGCUUUCC composition is detected.
