# Supplementary material for: Sequencing and Bioinformatics-Based Analyses of the microRNA Transcriptome in Hepatitis B–Related Hepatocellular Carcinoma
Source: PLoS One. 2011 Jan 25;6(1):e15304. doi: 10.1371/journal.pone.0015304 (PMC3026781; doi:10.1371/journal.pone.0015304)
Supplement: Figure S2 — RNA modifications of mature miR-122 and miR-21in the human liver. Sequence analyses of miR-122 (left column) and miR-21 (right column). The number of total (modified and nonmodified forms (A), modified forms (B), and individual modified sites (C) are indicated in the bar graph (DOC) [file pone.0015304.s002.doc]

**Figure S2**

**Figure S2.** RNA modifications of mature miR-122 and miR-21in the human liver. Sequence analyses of miR-122 (left column) and miR-21 (right column). The number of total (modified and nonmodified forms (**A**), modified forms (**B**), and individual modified sites (**C**) are indicated in the bar graph
