# Supplementary material for: Sequencing and Bioinformatics-Based Analyses of the microRNA Transcriptome in Hepatitis B–Related Hepatocellular Carcinoma
Source: PLoS One. 2011 Jan 25;6(1):e15304. doi: 10.1371/journal.pone.0015304 (PMC3026781; doi:10.1371/journal.pone.0015304)
Supplement: Table S1 — Clinical and pathologic characteristics of the study population and cells used. (DOC) [file pone.0015304.s003.doc]

**Supplementary Table S1.** Clinical and pathologic characteristics of the study population and cells used.

| No. | Age | Sex | | Infection | | | Tumor characteristics | | | | | | | | | | | | | | | | | Clinical course | |
| --- | --- | --- | --- | --- | --- | --- | --- | --- | --- | --- | --- | --- | --- | --- | --- | --- | --- | --- | --- | --- | --- | --- | --- | --- | --- |
|  |  |  |  |  |  |  | size　 (cm) | Stage (TMN) | | | | Pathological variables | | | | | | | | | | | | Recurrence (month) | Prognosis (month) |
|  |  |  |  | HBs  Ag | HBc　Ab | HCV 　Ab |  | T | N | M | Stage | Differentiation | Growth  type | fc | fc-  inf | sf | s | vp | vv | va | b | im | p |  |  |
| 1 | 70 | M | | + |  | - | 3 | 3 | 0 | 0 | 3a | moderately | ig | + | + | + | - | - | - | - | - | - | - | + (16) | Alive (57) |
| 2 | 68 | M | | + |  | - | 16 | 4 | 0 | 0 | 4a | well | ig | - | - | + | - | + | + | + | + | + |  | - (7) | Dead (7) |
| 3 | 64 | M | | + |  | - | 20 | 4 | 0 | 0 | 4a | poor | eg | + | + | + | - | + | + | + | + | + |  | + (5) | Alive (19) |
| 4 | 45 | M | | + |  | - | 10 | 4 | 1 | 0 | 4a | poorly | eg | - | + | + | - | + | + | + | - | + | - | - (7) | Dead (7) |
| 5 | 50 | M | | + |  | - | 3 | 2 | 0 | 0 | 2 | poor | eg | + | + | + | - | - | - | - | - | - |  | - (26) | Alive (26) |
| 6 | 48 | M | | + |  | - | 3 | 4 | 0 | 0 | 4a | poor | ig | + | + | + | - | + | + | + | - | + |  | + (3) | Alive (17) |
| 7 | 48 | M | | + |  | - | 12.5 | 3 | 0 | 0 | 3a | moderately | eg | + | + | + | - | + | + | - | - | - | - | + (6) | Dead (12) |
| 8 | 57 | M | | + |  | - | 4 | 4 | 0 | 0 | 4a | moderately | ig | - | - | - | - | + | + | + | + | + |  | + (2) | Dead (2) |
| 9 | 53 | M | | + |  | - | 5 | 4 | 0 | 0 | 4a | moderately | eg | + | + | + | + | + | + | - | - | + | - | + (1) | Dead (1) |
| 10 | 59 | M | | + |  | - | 7.7 | 3 | 0 | 0 | 3a | moderately | eg | - | + | + | - | + | + |  | - |  | - | Unknown (5) | Unknown (30) |
| 11 | 55 | M | | + |  | - | 1.3 | 2 | 0 | 0 | 2 | well | eg | + | + | + | - | - | - | - | - | - | - | + (25) | Alive (37) |
| 12 | 74 | M | | + |  | - | 7 | 4 | 0 | 0 | 4a | moderately | eg | + | + | + | + | + | - | - | - | + | - | - (8) | Dead (8) |
| 13 | 25 | M | | + |  | - | 3.5 | 4 | 0 | 0 | 4a | moderately | eg | + | - | + | - | + | + | - | - | - | - | Unknown (20) | Unknown (45) |
| 14 | 61 | F | | + |  | - | 3 | 2 | 0 | 0 | 2 | moderately | eg | + | + | + | - | + | + | - | - |  | - | - (60) | Alive (60) |
| 15 | 61 | M | | + |  | - | 8 | 4 | 0 | 0 | 4a | moderately | eg | - | - | - | + | + | + | + | - | + | - | + (12) | Dead (16) |
| 16 | 57 | M | | + |  | - | 3 | 2 | 0 | 0 | 2 | moderately | eg | + | + | + | - | - | - | - | - | - |  | - (89) | Alive (89) |
| 17 | 66 | M | | + |  | - | 4 | 3 | 0 | 0 | 3a | well | eg | + | + | + | - | + | + | - | - | - |  | + (7) | Alive (13) |
| 18 | 57 | M | | + |  | - | 3.5 | 2 | 0 | 0 | 2 | moderately | eg | + | + | + | - | - | - | - | - | - | - | - (64) | Alive (64) |
| 1* | 73 | F | | - | + | - | 2 | 1 | 0 | 0 | 1 | moderately | eg | + | - | + | - | - | - | - | - | - | - | Unknown (7) | Unknown (7) |
| 2* | 62 | M | | - | + | - | 10 | 4 | 0 | 0 | 4a | well | eg | + | + | + | - | + | + | + | + | + |  | - (8) | Alive (8) |
| 3* | 74 | M | | - | + | - | 8.5 | 3 | 0 | 0 | 3a | moderately | eg | + | + | + | - | + | + | - | - |  | - | - (57) | Alive (57) |
| 4* | 52 | M | | - | + | - | 3 | 3 | 0 | 0 | 3a | well | ig | + | + | + | - | + | - | - | - | - | - | - (90) | Alive (90) |
| HuH-1 | | | | + |  | - |  |  |  |  |  |  |  |  |  |  |  |  |  |  |  |  |  |  |  |
| JHH-7 | | | | + |  | - |  |  |  |  |  |  |  |  |  |  |  |  |  |  |  |  |  |  |  |
| PLC/PRF/5 | | | | + |  | - |  |  |  |  |  |  |  |  |  |  |  |  |  |  |  |  |  |  |  |
| C1 | 69 | | M | - |  | - | Colon^*^ |  |  |  |  |  |  |  |  |  |  |  |  |  |  |  |  |  |  |
| C2 | 62 | | M | - |  | - | Colon^*^ |  |  |  |  |  |  |  |  |  |  |  |  |  |  |  |  |  |  |
| C3 | 77 | | F | - |  | - | Colon^*^ |  |  |  |  |  |  |  |  |  |  |  |  |  |  |  |  |  |  |
| C4 | 65 | | M | - |  | - | Stomach^*^ |  |  |  |  |  |  |  |  |  |  |  |  |  |  |  |  |  |  |
| C5 | 65 | | M | - |  | - | Colon^*^ |  |  |  |  |  |  |  |  |  |  |  |  |  |  |  |  |  |  |
| C6 | 65 | | M | - |  | - | Rectum^*^ |  |  |  |  |  |  |  |  |  |  |  |  |  |  |  |  |  |  |

^*^Organ of primary cancer. fc, capsule formation; fc-inf, capsule invasion; sf, septum formation; s, serosa invasion; vp, portal vein invasion; vv, venous invasion; a, arterial invasion; b, bile duct invasion; im, intrahepatic metastasis; eg, expansive growth; ig, invasive growth
