# Supplementary material for: Sequencing and Bioinformatics-Based Analyses of the microRNA Transcriptome in Hepatitis B–Related Hepatocellular Carcinoma
Source: PLoS One. 2011 Jan 25;6(1):e15304. doi: 10.1371/journal.pone.0015304 (PMC3026781; doi:10.1371/journal.pone.0015304)
Supplement: Table S2 — Primers used for the PCR-based small microRNA detection. (DOC) [file pone.0015304.s004.doc]

**Supplementary Table S2 Primers used for the PCR-based small microRNA detection.**

| Name (Accession  No.) | Sequence (5’-3’ |
| --- | --- |
| AB372577 | CCCCAGATTCCACACC |
| AB372592 | GCCGCTGGTGCTGCGACTGC |
| AB372613 | CCTTGGAGGCCTGGCTTTGTGAT |
| AB372624 | CTGTGGTAGTGAAAAGTCTGT |
| AB372665 | CCAGAAACGAGTGAGTC |
| AB372673 | CAGGGGGCAGGGAGGGC |
| AB372682 | CCCACCTGAGTGCCGTGAGC |
| AB372692 | CATTTTAGCTGGAAGATGC |
| AB372776 | TGGAAAGGATGATACACA |
| AB372777 | GTGGTGGTGGTGGGGGGG |
| GAPDH | ACAGTCAGCCGCATCTTCTT |
