# Supplementary material for: Sequencing and Bioinformatics-Based Analyses of the microRNA Transcriptome in Hepatitis B–Related Hepatocellular Carcinoma
Source: PLoS One. 2011 Jan 25;6(1):e15304. doi: 10.1371/journal.pone.0015304 (PMC3026781; doi:10.1371/journal.pone.0015304)
Supplement: Table S3 — Summary of the cloned small RNAs. (DOC) [file pone.0015304.s005.doc]

**Supplementary Table S3. Summary of the cloned small RNAs.**

| Category | 454 sequencer | |  |  |  |  |  |  |  |  |  |  |  |  |  |  |  |  |  |  |  |  |
| --- | --- | --- | --- | --- | --- | --- | --- | --- | --- | --- | --- | --- | --- | --- | --- | --- | --- | --- | --- | --- | --- | --- |
|  | N | T |  |  |  |  |  |  |  |  |  |  |  |  |  |  |  |  |  |  |  |  |
| Reads | 268708 | 314359 |  |  |  |  |  |  |  |  |  |  |  |  |  |  |  |  |  |  |  |  |
| Target miRNA | 208818 | 257402 |  |  |  |  |  |  |  |  |  |  |  |  |  |  |  |  |  |  |  |  |
| Other miRNA | 922 | 1508 |  |  |  |  |  |  |  |  |  |  |  |  |  |  |  |  |  |  |  |  |
| Genome | 2884 | 2712 |  |  |  |  |  |  |  |  |  |  |  |  |  |  |  |  |  |  |  |  |
| No hit genome | 35493 | 35170 |  |  |  |  |  |  |  |  |  |  |  |  |  |  |  |  |  |  |  |  |
| mRNA | 7279 | 6053 |  |  |  |  |  |  |  |  |  |  |  |  |  |  |  |  |  |  |  |  |
| piRNA | 1440 | 2983 |  |  |  |  |  |  |  |  |  |  |  |  |  |  |  |  |  |  |  |  |
| rRNA | 10161 | 5474 |  |  |  |  |  |  |  |  |  |  |  |  |  |  |  |  |  |  |  |  |
| snRNA | 343 | 700 |  |  |  |  |  |  |  |  |  |  |  |  |  |  |  |  |  |  |  |  |
| snoRNA | 747 | 654 |  |  |  |  |  |  |  |  |  |  |  |  |  |  |  |  |  |  |  |  |
| tRNA | 621 | 1703 |  |  |  |  |  |  |  |  |  |  |  |  |  |  |  |  |  |  |  |  |
|  |  |  |  |  |  |  |  |  |  |  |  |  |  |  |  |  |  |  |  |  |  |  |
| Category | Conventional cloning | | | | | | | | | | | | | | | | | | | | | |
|  | N1 | N2 | N3 | N4 | N5 | N6 | N7 | N8 | N9 | N10 | N11 | N12 | N13 | N14 | N15 | N16 | N17 | N18 | N*1 | N*2 | N*3 | N*4 |
| Reads | 1517 | 1132 | 895 | 1119 | 1341 | 1311 | 1140 | 1443 | 1083 | 961 | 637 | 1117 | 1097 | 771 | 1160 | 1399 | 1319 | 808 | 1235 | 1286 | 1315 | 1152 |
| Target miRNA | 1228 | 988 | 620 | 966 | 971 | 1180 | 638 | 1297 | 933 | 521 | 500 | 585 | 930 | 427 | 982 | 1191 | 1060 | 706 | 664 | 1162 | 1072 | 990 |
| Other miRNA | 6 | 7 | 7 | 2 | 0 | 0 | 1 | 9 | 3 | 6 | 0 | 3 | 1 | 4 | 12 | 15 | 5 | 1 | 11 | 0 | 10 | 1 |
| Genome | 13 | 5 | 12 | 6 | 26 | 3 | 54 | 2 | 9 | 29 | 8 | 16 | 6 | 15 | 3 | 7 | 7 | 4 | 21 | 8 | 2 | 7 |
| No hit genome | 202 | 108 | 182 | 123 | 241 | 96 | 320 | 106 | 120 | 207 | 85 | 387 | 61 | 164 | 128 | 141 | 182 | 63 | 303 | 91 | 178 | 129 |
| mRNA | 12 | 7 | 15 | 8 | 70 | 21 | 91 | 6 | 4 | 60 | 12 | 104 | 16 | 33 | 9 | 4 | 14 | 11 | 25 | 11 | 15 | 7 |
| piRNA | 12 | 1 | 25 | 2 | 10 | 1 | 7 | 4 | 3 | 24 | 10 | 5 | 5 | 30 | 5 | 3 | 8 | 1 | 46 | 4 | 4 | 4 |
| rRNA | 37 | 11 | 25 | 10 | 9 | 9 | 18 | 14 | 7 | 88 | 14 | 10 | 76 | 72 | 13 | 22 | 35 | 18 | 70 | 4 | 21 | 11 |
| snRNA | 3 | 2 | 4 | 1 | 2 | 0 | 1 | 2 | 3 | 12 | 4 | 1 | 0 | 13 | 3 | 6 | 1 | 1 | 16 | 0 | 10 | 2 |
| snoRNA | 1 | 1 | 1 | 0 | 1 | 0 | 2 | 0 | 0 | 2 | 0 | 3 | 2 | 0 | 1 | 1 | 3 | 2 | 4 | 0 | 1 | 1 |
| tRNA | 3 | 2 | 4 | 1 | 11 | 1 | 8 | 3 | 1 | 12 | 4 | 3 | 0 | 13 | 4 | 9 | 4 | 1 | 75 | 6 | 2 | 0 |
|  |  |  |  |  |  |  |  |  |  |  |  |  |  |  |  |  |  |  |  |  |  |  |
| Category | Conventional cloning | | | | | | | | | | | | | | | | | | | | | |
|  | T1 | T2 | T3 | T4 | T5 | T6 | T7 | T8 | T9 | T10 | T11 | T12 | T13 | T14 | Ｔ15 | T16 | T17 | T18 | T*1 | T*2 | T*3 | T*4 |
| Reads | 1297 | 1184 | 1250 | 1388 | 1395 | 1252 | 1218 | 1055 | 1223 | 850 | 1014 | 810 | 1015 | 900 | 1352 | 1225 | 1361 | 754 | 1270 | 1179 | 1456 | 1320 |
| Target miRNA | 659 | 991 | 909 | 1050 | 940 | 1081 | 889 | 965 | 995 | 583 | 516 | 554 | 953 | 622 | 1157 | 1046 | 1109 | 669 | 852 | 907 | 1156 | 1150 |
| Other miRNA | 11 | 12 | 12 | 15 | 2 | 1 | 3 | 6 | 6 | 4 | 2 | 0 | 2 | 3 | 13 | 12 | 2 | 2 | 1 | 0 | 12 | 5 |
| Genome | 76 | 3 | 15 | 29 | 25 | 3 | 7 | 1 | 7 | 18 | 27 | 4 | 0 | 22 | 2 | 6 | 13 | 2 | 52 | 6 | 9 | 1 |
| No hit genome | 287 | 155 | 272 | 259 | 360 | 104 | 135 | 62 | 195 | 147 | 272 | 213 | 36 | 112 | 148 | 115 | 162 | 67 | 232 | 163 | 206 | 150 |
| mRNA | 50 | 0 | 8 | 4 | 41 | 44 | 144 | 7 | 0 | 11 | 56 | 26 | 1 | 21 | 2 | 2 | 20 | 3 | 52 | 84 | 19 | 1 |
| piRNA | 59 | 0 | 10 | 3 | 5 | 2 | 10 | 3 | 2 | 28 | 27 | 7 | 7 | 46 | 8 | 1 | 5 | 2 | 29 | 12 | 8 | 1 |
| rRNA | 83 | 16 | 16 | 12 | 14 | 8 | 19 | 8 | 9 | 32 | 51 | 2 | 11 | 34 | 14 | 37 | 42 | 3 | 39 | 2 | 29 | 7 |
| snRNA | 12 | 6 | 2 | 15 | 0 | 0 | 0 | 0 | 6 | 8 | 39 | 0 | 0 | 7 | 5 | 3 | 3 | 0 | 0 | 0 | 12 | 1 |
| snoRNA | 18 | 0 | 1 | 0 | 3 | 3 | 9 | 2 | 0 | 6 | 5 | 1 | 2 | 2 | 0 | 0 | 0 | 2 | 6 | 1 | 0 | 0 |
| tRNA | 42 | 1 | 5 | 1 | 5 | 6 | 2 | 1 | 3 | 13 | 19 | 3 | 3 | 31 | 3 | 3 | 5 | 4 | 7 | 4 | 5 | 4 |
|  |  |  |  |  |  |  |  |  |  |  |  |  |  |  |  |  |  |  |  |  |  |  |
| Category | Conventional cloning | | | | | | | | |  |  |  |  |  |  |  |  |  |  |  |  |  |
|  | C1 | C2 | C3 | C4 | C5 | C6 | JHH | HuH1 | PLC |  |  |  |  |  |  |  |  |  |  |  |  |  |
| Reads | 1185 | 1467 | 1400 | 1466 | 1389 | 1315 | 1171 | 1319 | 1185 |  |  |  |  |  |  |  |  |  |  |  |  |  |
| Target miRNA | 986 | 1278 | 1202 | 1261 | 1240 | 1168 | 913 | 1023 | 910 |  |  |  |  |  |  |  |  |  |  |  |  |  |
| Other miRNA | 10 | 8 | 6 | 14 | 3 | 7 | 1 | 5 | 5 |  |  |  |  |  |  |  |  |  |  |  |  |  |
| Genome | 8 | 1 | 4 | 8 | 8 | 5 | 6 | 9 | 5 |  |  |  |  |  |  |  |  |  |  |  |  |  |
| No hit genome | 155 | 119 | 129 | 156 | 108 | 105 | 192 | 234 | 219 |  |  |  |  |  |  |  |  |  |  |  |  |  |
| mRNA | 8 | 4 | 21 | 2 | 9 | 4 | 5 | 6 | 6 |  |  |  |  |  |  |  |  |  |  |  |  |  |
| piRNA | 7 | 9 | 6 | 3 | 5 | 5 | 3 | 5 | 6 |  |  |  |  |  |  |  |  |  |  |  |  |  |
| rRNA | 11 | 35 | 23 | 13 | 10 | 14 | 43 | 32 | 30 |  |  |  |  |  |  |  |  |  |  |  |  |  |
| snRNA | 0 | 6 | 7 | 7 | 1 | 4 | 4 | 2 | 0 |  |  |  |  |  |  |  |  |  |  |  |  |  |
| snoRNA | 0 | 0 | 0 | 0 | 2 | 1 | 3 | 0 | 2 |  |  |  |  |  |  |  |  |  |  |  |  |  |
| tRNA | 0 | 7 | 2 | 2 | 3 | 2 | 1 | 3 | 2 |  |  |  |  |  |  |  |  |  |  |  |  |  |

N, a sample from the adjacent normal liver; T, hepatocellular carcinoma; C, control; N* and T*, samples from the livers of patients having historic infection with HBV.
