# Supplementary material for: Sequencing and Bioinformatics-Based Analyses of the microRNA Transcriptome in Hepatitis B–Related Hepatocellular Carcinoma
Source: PLoS One. 2011 Jan 25;6(1):e15304. doi: 10.1371/journal.pone.0015304 (PMC3026781; doi:10.1371/journal.pone.0015304)
Supplement: Table S5 — Summary of predicted novel miRNAs. The predicted novel miRNAs with their PhastCon scores are listed. The most frequent cloned sequences, genome locations, clone counts, and conservation scores calculated with PhastCon are listed. The precursor structures are listed in Fig. 1. (DOC) [file pone.0015304.s007.doc]

**Supplementary Table S5**

Summary of predicted novel miRNAs. The predicted novel miRNAs with their PhastCon scores are listed. The most frequent cloned sequences, genome locations, clone counts, and conservation scores calculated with PhastCon are listed. The precursor structures are listed in Fig. 1.

| **Accession Number** | **Sequence** | **Strand** | **Chromosome** | **Mature miRNA** | | | **Precursor miRNA** | | | | | | | **Total clone counts** |
| --- | --- | --- | --- | --- | --- | --- | --- | --- | --- | --- | --- | --- | --- | --- |
|  |  |  |  | **Start** | **End** | **PhastCon score** | **Start** | **End** | **PhastCon score** | **dG kcal/mol** | **Pos. from loop** | **Max. loop** | **Max. internal loop** |  |
| **Novel miRNAs** | | | | | | | | | | | | | | |
| AB372577 | CCCCAGATTCCACACC | + | 1 | 154326366 | 154326381 | 1.00 | 154326362 | 154326427 | 0.85 | -28.9 | 11 | 4 | 7 | 1 |
| AB372592 | GCCGCTGGTGCTGCGACTGC | - | 1 | 37991420 | 37991439 | 0.98 | 37991387 | 37991496 | 0.98 | -58.0 | 3 | 3 | 5 | 2 |
| AB372613 | CCTTGGAGGCCTGGCTTTGTGAT | + | 12 | 11707789 | 11707811 | 0.90 | 11707784 | 11707903 | 0.95 | -41.1 | 29 | 4 | 5 | 1 |
| AB372624 | CTGTGGTAGTGAAAAGTCTGT | + | 13 | 90801523 | 90801543 | 0.97 | 90801437 | 90801546 | 0.95 | -41.4 | 25 | 13 | 6 | 3 |
| AB372665 | CCAGAAACGAGTGAGTC | - | 16 | 68845316 | 68845332 | 0.98 | 68845272 | 68845346 | 0.81 | -24.7 | 4 | 6 | 8 | 1 |
| AB372673 | CAGGGGGCAGGGAGGGC | + | 6 | 31720135 | 31720151 | 1.00 | 31720129 | 31720248 | 0.99 | -30.9 | 36 | 7 | 10 | 1 |
| AB372682 | CCCACCTGAGTGCCGTGAGC | + | 19 | 3501977 | 3501996 | 0.81 | 3501972 | 3502050 | 0.88 | -38.1 | 9 | 8 | 7 | 1 |
| AB372692 | CATTTTAGCTGGAAGATGC | + | 2 | 17861039 | 17861057 | 1.00 | 17861030 | 17861139 | 0.91 | -27.1 | 28 | 4 | 4 | 1 |
| AB372776 | TGGAAAGGATGATACACA | - | 12 | 101297625 | 101297642 | 1.00 | 101297619 | 101297728 | 0.99 | -20.9 | 24 | 6 | 7 | 1 |
| AB372777 | GTGGTGGTGGTGGGGGGG | - | 12 | 120727042 | 120727059 | 1.00 | 120726975 | 120727084 | 0.97 | -54.8 | 8 | 7 | 10 | 1 |
| **Novel antisense miRNAs** | | | | | | | | | | | | | | |
| AB372579 | CACCCAGATCTGCGGCCTAATCA | + | 1 | 169337504 | 169337526 | 0.03 | 169337477 | 169337586 | 0.02 | -53.7 | 7 | 5 | 5 | 1 |
| AB372768 | ATAGGCCACGGATCTGGGCAA |  |  | 169337543 | 169337563 | 0.00 |  |  |  |  |  |  |  | 1 |
| AB372588 | CAGCACTGTCCGGTAAGATGCT | - | 20 | 1093119 | 1093140 | 1.00 | 1093107 | 1093194 | 0.87 | -53.7 | 4 | 5 | 5 | 1 |
