# Supplementary material for: Sequencing and Bioinformatics-Based Analyses of the microRNA Transcriptome in Hepatitis B–Related Hepatocellular Carcinoma
Source: PLoS One. 2011 Jan 25;6(1):e15304. doi: 10.1371/journal.pone.0015304 (PMC3026781; doi:10.1371/journal.pone.0015304)
Supplement: Table S6 — Summary of novel opposite miRNAs. The novel opposite miRNAs with their precursor predicted structures, chromosomal locations, and number of clone reads are listed. The most frequent cloned sequence is listed, and the sequence that does not match the genomic sequence is shown in green. Comparison of the expression levels between miRNAs from the 3′-arm and 5′-arm are calculated with the paired Student's t-test. (DOC) [file pone.0015304.s008.doc]

**Supplementary Table S6** Summary of novel opposite miRNAs. The novel opposite miRNAs with their precursor predicted structures, chromosomal locations, and number of clone reads are listed. The most frequent cloned sequence is listed, and the sequence that does not match the genomic sequence is shown in green. Comparison of the expression levels between miRNAs from the 3′-arm and 5′-arm are calculated with the paired Student’s *t*-test.

| Opposite miRNA sequence | Precursor sequence:  *Red*, known miRNA; *Blue*, opposite miRNA | Genome　context | Number　　of reads |
| --- | --- | --- | --- |
| hsa-miR-103-1-5p  AB372800  GGCTTCTTTACAGTGCTGCCTTGT | UAC C -- U U C U G A  UGCC UC GGCU CU UACAGUGCUGC UUG U C U  ACGG AG UCGG GA AUGUUACGACG AAC A G A  GUU A UA - C - U G U | 5〔-〕  167920523-  167920546 | *HCC* 3  *ANL* 0 |
| hsa-miR-210-5p  AB372802  AGCCCCTGCCCACCGCACACTGC | ACCC CA -C GG C CC - C C -  GG GUGC UCCAGGCGCAG CAGCC CUG CAC CGCACA UG G CUGC  CC CGCG GGGUCCGUGUC GUCGG GAC GUG GCGUGU AC C GACC C  ---C AG AC UA C -A U C C A | 11〔-〕  558149-  558171 | *HCC* 0  *ANL* 3 |
| hsa-miR-660-3p  AB372803  ACCTCCTGTGTGCATGGATTACA | ----------CU C -CAUAC U C U GAAUU  GCUC UUCUCC CCAU GCAUAU GGAG UGU  CGAG GGGAGG GGUA CGUGUG CCUC ACA C  GUGCUACUGUUC U ACAUUA - U C AAACU | X〔+〕  49664640-  49664662 | *HCC* 4  *ANL* 3 |
| hsa-miR-98-3p  AB372804  CTATACAACTTACTACTTTCCT | A UC - U U --------- AGGGA  GGAU UGCU CAUGCCAGGG GAGGUAGUAAGUUGUAU GUUG UGGGGU  CUUA ACGG GUGUGGUCCC UUUCAUCAUUCAACAUA CAAU ACCCCG U  A -U U - U AGAAGAUUA GAUUA | X〔-〕 53599928-  53599948 | *HCC* 3  *ANL* 0 |
| hsa-miR-487a-5p  AB372806  GTGGTTATCCCTGCTGTGT | U G UUA C - C A  GGUACU GAAGA UGG UCCCUG UGUG UUCG UUA U  CUAUGA UUUUU ACC AGGGAC AUAC AAGC AGU UU  C G UAC - U - A | 14〔+〕  100588548-  100588566 | *HCC* 0  *ANL* 3 |
| hsa-mir-552-5p  AB372807  TGTTTAACCTTTTGCCTGTTGG | AACCAUUCAA UU UG UU AA  AUAUACCACAGUUUGUUUAACC U CCUGUUGG G G UAUAUGGUGUCAAACAGAUUGG A GGACAACU C U A  -------ACA UC GU UU CG | 1  34907837-  34907858  〔-〕 | *HCC* 2  *ANL* 1 |
| hsa-mir-383-3p  AB372808  CCACAGCACTGCCTGGTCA | -C A AA A UUG GGA  CUC UC GAUCAG GGUG UUGUGGCU GGU U  GAG AG CUGGUC UCAC GACACCGA CUA A  AA A CG - --- AUU | 8〔-〕  14755325-  14755343 | *HCC* 0  *ANL* 1 |
| hsa-miR-579-5p  AB372809  TCGCGGTTTGTGCCAGATGA | CAUAUUAG A CG AA  GUU AUGCAAAAGUAAUCGCGGUUUGUGCCAGAUGA AUUUG U  CAA UACGUUUUUAUUAGCGCCAAAUAUGGUUUACU UAAAU U  -------- C -- AA | 5〔-〕  32430314-  32430295 | *HCC* 1  *ANL* 0 |
| hsa-miR-181b-1-3p  AB372810  CTCACTGAACAATGAATGCA | CCUGUGCAGAGAUUAUUUUUUAAAA AUCAA CUG GAA G  GGUCACA CAUUCAUUG UCGGUGGGUU CU U  CCGGUGU GUAAGUAAC AGUCACUCGA GG G  -------------------UUCGCC -CAAC --A ACA U | 1〔-〕  197094640-  197094659 | *HCC* 0  *ANL* 1 |
| hsa-miR-520e-5p  AB372812  CTCAAGATGGAAGCAGTTTCT | CU U U G GUUG G  UCUC GC GUGACCCUCAAGA GGAAGCA UUUCU UCU A  AGAG UG CAUUGGGAGUUUU CCUUCGU AAAGA AGG A  UU U U G ---A A | 19〔+〕  58870793-  58870813 | *HCC* 1  *ANL* 0 |
| hsa-mir-496-5p  AB372814  CCCAAGTCAGGTACTCGAATGGA | -----UCA U U U GU UUA  CCCAAG GGUAC CGAA GGAGGUUG CCAUG GUGUUCAUU U  GGGUUC UCAUG GCUU CCUCUAAC GGUAC UAUGAGUAG U  UUCUUAAC - U C AU UAU | 14〔+〕  100596663-  100596685 | *HCC* 1  *ANL* 0 |
| has-miR-518b-5p  CCAGAGGGAAGCGCTTTCTGA | U GC C A UC G U  UCA GCUGUG CCUC AGAGGG AGCGCUU UGUU UC G  AGU UGGCAU GGAG UUUCCC UCGCGAA ACAA AG A A  U UU A C -- A A | 19〔+〕  5420421-  5420440 | *HCC* 87  *ANL* 106 |
| hsa-miR-519a-2-5p  CCTCTACAGGGAAGCGCTTTCT | U U C C A GUUG U  UCUCAGGC GUG C CUCUA AGGGA GCGCUUUCU UC G  AGAGUUUG CAU G GAGAU UUCCU CGUGAAAGG AG A A  U U U U A --AA A | 19〔+〕  54265612-  54265633 | *HCC* 2  *ANL* 1 |
| hsa-mir-519d-5p  CCTCCAAAGGGAAGCGCTTTCTGT | U U C C A UC UUU  UCCCA GC GUGAC CUC AAAGGGA GCGCUU UGUUUG U  AGGGU UG CAUUG GAG UUUCCCU CGUGAA ACAAAU C  U C U A C -- UCU | 19〔+〕  54216615-  54266638 | *HCC* 11  *ANL* 1 |
| hsa-mir-1258-5p  CCACGACCTAATCCTAACTCC | -- UGCG  CUGUGGCUUCCACGACCUAAUCCUAACUCC A  GACACCGAAGGUGCUGGAUUAGGAUUGAGG U G  AG UCCC | 2〔-〕  180725506-  180725626 | *HCC* 2  *ANL* 0 |
