# Supplementary material for: Sequencing and Bioinformatics-Based Analyses of the microRNA Transcriptome in Hepatitis B–Related Hepatocellular Carcinoma
Source: PLoS One. 2011 Jan 25;6(1):e15304. doi: 10.1371/journal.pone.0015304 (PMC3026781; doi:10.1371/journal.pone.0015304)
Supplement: Table S7 — Sequence compositions of mature has-miR21 and miR122. Multiple sequence alignments were generated with the CLUSTALW program. Modified nucleotides at positions +1 to +18 are depicted in color [red, A→I (G); yellow, T→C; blue, T→A; green, C→T; pink, G→A] Nucleotides that have no modified sequences are colored black (100%), dark-gray (80–99%), and light-gray (60–79%). The gray colorations indicate the minus or plus position of the registered sequence. Note that there are particular modified patterns according to the particular nucleotide. (DOC) [file pone.0015304.s009.doc]

**Supplementary Table S7.** Sequence compositions of mature has-miR21 and miR122. Multiple sequence alignments were generated with the CLUSTALW program. Modified nucleotides at positions +1 to +18 are depicted in color [red, A→I (G); yellow, T→C; blue, T→A; green, C→T; pink, G→A] Nucleotides that have no modified sequences are colored black (100%), dark-gray (80-99%), and light-gray (60-79%). The gray colorations indicate the minus or plus position of the registered sequence. Note that there are particular modified patterns according to the particular nucleotide.

| Sequence miR-21 | | Reads | | |
| --- | --- | --- | --- | --- |
| Rank (Total Reads) | +1 +18 | HCC | ANL | Total |
| Rank-10 | **--TA**G**CCTATCAGACTGATGTTGA-----** | 119 | 62 | 181 |
| Rank-30 | **--TA**GC**CTATCAGACTGATGTTGAC----** | 64 | 39 | 103 |
| Rank-45 | **--TA**GC**ATATCAGACTGATGTTGA-----** | 27 | 12 | 39 |
| Rank-1 | **--TA**GC**TTATCAGACTGATGTTGA-----** | 26 386 | 15 664 | 42 050 |
| Rank-43 | **--TA**GC**TTATCAGACTGATGTTGAT----** | 23 | 19 | 42 |
| Rank-5 | **--TA**GC**TTATCAGACTGATGTTGAA----** | 379 | 128 | 507 |
| Rank-40 | **--TA**GC**TTATCAGACTGATATTGA-----** | 36 | 8 | 44 |
| Rank-34 | **-ATA**GC**TTATCAGACTGATGTTGA-----** | 46 | 16 | 62 |
| Rank-50 | **GATA**GC**TTATCAGACTGATGTTGA-----** | 20 | 14 | 34 |
| Rank-39 | **--TA**GC**TTATTAGACTGATGTTGA-----** | 30 | 17 | 47 |
| Rank-38 | **-GTA**GC**TTATCAGACTGATGTTGA-----** | 40 | 9 | 49 |
| Rank-41 | **--TA**GC**TTATCAGATTGATGTTGA-----** | 24 | 19 | 43 |
| Rank-11 | **--TA**GC**TTATCAGACTGATGTTGG-----** | 120 | 54 | 174 |
| Rank-32 | **--TA**GC**TTATCAGACTGATGTTGGC----** | 44 | 32 | 76 |
| Rank-4 | **--TA**GC**TTATCAGACTGATGTTG------** | 504 | 300 | 804 |
| Rank-6 | **--TA**GC**TTATCAGACTGATGTTGT-----** | 157 | 135 | 292 |
| Rank-8 | **--TA**GC**TTATCAGACTGATGTTGACT---** | 135 | 60 | 195 |
| Rank-48 | **--TA**GC**TTATCAGACTGATGTTGACTGTA** | 30 | 4 | 34 |
| Rank-20 | **--TA**GC**TTATCAGACTGATG---------** | 89 | 46 | 135 |
| Rank-35 | **--TA**GC**TTATCAGACTGATGCTGAC----** | 39 | 22 | 61 |
| Rank-44 | **--TA**GC**TTATCAGACTGATGTTAA-----** | 20 | 19 | 39 |
| Rank-19 | **--TA**GC**TCATCAGACTGATGTTGA-----** | 79 | 65 | 144 |
| Rank-22 | **--TA**GC**TCATCAGACTGATGTTGAC----** | 73 | 55 | 128 |
| Rank-49 | **--TA**GC**TAATCAGACTGATGTTGA-----** | 23 | 11 | 34 |
| Rank-37 | **--TA**GC**TTGTCAGACTGATGTTGA-----** | 43 | 14 | 57 |
| Rank-42 | **--TA**GC**TTGTCAGACTGATGTTGAC----** | 31 | 11 | 42 |
| Rank-14 | **--TA**GC**TTATCGGACTGATGTTGA-----** | 103 | 58 | 161 |
| Rank-31 | **--TA**GC**TTATCGGACTGATGTTGAC----** | 62 | 34 | 96 |
| Rank-47 | **-ATA**GC**TTATCAGACTGATGTTGAC----** | 24 | 13 | 37 |
| Rank-18 | **--TA**GC**TTATCAGACCGATGTTGA-----** | 102 | 46 | 148 |
| Rank-23 | **--TA**GC**TTATCAGACCGATGTTGAC----** | 76 | 48 | 124 |
| Rank-7 | **--TA**GC**TTATCAGACTGATGTTGACC---** | 180 | 85 | 265 |
| Rank-9 | **--TA**GC**TTATCAGACTGATGTTGACCC--** | 126 | 68 | 194 |
| Rank-13 | **--TA**GC**TTACCAGACTGATGTTGAC----** | 110 | 60 | 170 |
| Rank-21 | **--TA**GC**TTACCAGACTGATGTTGA-----** | 63 | 70 | 133 |
| Rank-46 | **--TA**GC**TTAACAGACTGATGTTGAC----** | 21 | 17 | 38 |
| Rank-2 | **--TA**GC**TTATCAGACTGATGTTGAC----** | 17 191 | 10 075 | 27 266 |
| Rank-24 | **--TA**GC**TTATCAGACTGACGTTGA-----** | 81 | 35 | 116 |
| Rank-25 | **--TA**GC**TTATCAGACTGACGTTGAC----** | 61 | 54 | 115 |
| Rank-12 | **--TG**GC**TTATCAGACTGATGTTGA-----** | 102 | 70 | 172 |
| Rank-29 | **--TG**GC**TTATCAGACTGATGTTGAC----** | 60 | 45 | 105 |
| Rank-3 | **--TA**GC**TTATCAGACTGATGTTGACA---** | 616 | 252 | 868 |
| Rank-15 | **--TA**GC**TTATCAGGCTGATGTTGA-----** | 90 | 70 | 160 |
| Rank-17 | **--TA**GC**TTATCAGGCTGATGTTGAC----** | 88 | 63 | 151 |
| Rank-28 | **--TA**GC**TTATCAGACTGGTGTTGA-----** | 75 | 32 | 107 |
| Rank-33 | **--TA**GC**TTATCAGACTGGTGTTGAC----** | 41 | 30 | 71 |
| Rank-16 | **--CA**GC**TTATCAGACTGATGTTGA-----** | 88 | 72 | 160 |
| Rank-27 | **--CA**GC**TTATCAGACTGATGTTGAC----** | 64 | 46 | 110 |
| Rank-26 | **-TAA**GC**TTATCAGACTGATGTTGA-----** | 65 | 49 | 114 |
| Rank-36 | -TAAGCTTATCA**G**ACT**G**ATGTTGAC---- | 33 | 25 | 58 |
|  | |  | | |
| Sequence miR-122 | | Reads | | |
| Rank (Total Reads) | **+1 +18** | HCC | ANL | Total |
| Rank-3 | -TGGAGTGTGACAATGGTGTTTGT-- | 6 675 | 14 639 | 21 314 |
| Rank-15 | -TGGAGTGTGACAATGGTGTTTGTT- | 80 | 179 | 259 |
| Rank-27 | -TGGAGTGTGACAATGGTGTTTGTA- | 47 | 112 | 159 |
| Rank-22 | -TGGAGTGTGACAATGGTGTTTGTCC | 53 | 144 | 197 |
| Rank-33 | -TGGAGTGTGACAAT**A**GTGTTTG--- | 21 | 97 | 118 |
| Rank-1 | -TGGAGTGTGACAATGGTGTTTG--- | 19 486 | 25 790 | 45 276 |
| Rank-25 | -TGGAGTGTGACA**G**TGGTGTTTG--- | 70 | 100 | 170 |
| Rank-42 | -TGGAGTGTGACA**G**TGGTGTTTGA-- | 29 | 59 | 88 |
| Rank-49 | -TGGAGTGTGACAATGGTGTTTGAC- | 25 | 55 | 80 |
| Rank-36 | -TGGAGTGTGAC**G**ATGGTGTTTG--- | 31 | 73 | 104 |
| Rank-43 | -TGGAGTGTGAC**G**ATGGTGTTTGA-- | 26 | 61 | 87 |
| Rank-2 | -TGGAGTGTGACAATGGTGTTTGA-- | 11 557 | 22 710 | 34 267 |
| Rank-18 | -TGGAGTGTGACAATGGTGTTTGAT- | 70 | 151 | 221 |
| Rank-26 | -TGGAGTGTGACAA**C**GGTGTTTG--- | 60 | 101 | 161 |
| Rank-37 | -TGGAGTGTGACAA**C**GGTGTTTGA-- | 27 | 76 | 103 |
| Rank-20 | -TGGAGTG**C**GACAATGGTGTTTG--- | 102 | 107 | 209 |
| Rank-21 | -TGGAGTG**C**GACAATGGTGTTTGA-- | 65 | 137 | 202 |
| Rank-12 | -TGGAGTGTGACAATGGTGTTTGAA- | 101 | 236 | 337 |
| Rank-17 | -TGGAGTGTG**G**CAATGGTGTTTG--- | 112 | 123 | 235 |
| Rank-40 | -TGGAGTGTG**G**CAATGGTGTTTGT-- | 34 | 61 | 95 |
| Rank-19 | -TGGAGTGTG**G**CAATGGTGTTTGA-- | 57 | 159 | 216 |
| Rank-48 | -TGGAGTGTG**G**CAATGGTGTTT---- | 33 | 50 | 83 |
| Rank-29 | -TGGAGTGTGACAATGGTGTTTGA-- | 84 | 61 | 145 |
| Rank-13 | -**C**GGAGTGTGACAATGGTGTTTGA-- | 120 | 154 | 274 |
| Rank-24 | -**C**GGAGTGTGACAATGGTGTTTG--- | 90 | 103 | 193 |
| Rank-44 | --GGAGTGTGACAATGGTGTTTGA-- | 21 | 66 | 87 |
| Rank-45 | --GGAGTGTGACAATGGTGTTTG--- | 41 | 46 | 87 |
| Rank-46 | aTGGAGTGTGACAATGGTGTTTG--- | 37 | 49 | 86 |
| Rank-47 | aTGGAGTGTGACAATGGTGTTTGA-- | 14 | 72 | 86 |
| Rank-28 | -TGGAGTGTGACAATGG**C**GTTTG--- | 56 | 90 | 146 |
| Rank-50 | -TGGAGTGTGACAATGG**C**GTTTGT-- | 21 | 54 | 75 |
| Rank-32 | -TGGAGTGTGACAATGG**C**GTTTGA-- | 20 | 102 | 122 |
| Rank-14 | -TGGAG**C**GTGACAATGGTGTTTG--- | 113 | 153 | 266 |
| Rank-41 | -TGGAG**C**GTGACAATGGTGTTTGT-- | 33 | 56 | 89 |
| Rank-23 | -TGGAG**C**GTGACAATGGTGTTTGA-- | 62 | 133 | 195 |
| Rank-10 | -TGGAGTGTGACAATGGTGTTTGG-- | 127 | 213 | 340 |
| Rank-39 | -T**A**GAGTGTGACAATGGTGTTTG--- | 50 | 47 | 97 |
| Rank-5 | -TGGAGTGTGACAATGGTGTT----- | 461 | 972 | 1,433 |
| Rank-38 | -TGGAGTGTGACAATGGTGTTTCC-- | 44 | 53 | 97 |
| Rank-6 | -TGGAGTGTGACAATGGTGTTT**A**--- | 484 | 721 | 1,205 |
| Rank-30 | -TGGAGTGTGACAATGGTGTTT**A**T-- | 27 | 110 | 137 |
| Rank-11 | -TGGAGTGTGACAATGGTGTTT**A**A-- | 104 | 235 | 339 |
| Rank-7 | -TGGAGTGTGACAATGGTGTTA---- | 202 | 437 | 639 |
| Rank-16 | -TGGAGTGTGACAATGGTGTTAA--- | 77 | 180 | 257 |
| Rank-34 | -TGGAGTGTGACAATGGTGTTGA--- | 56 | 53 | 109 |
| Rank-35 | -TGGAGTGTGACAATGGTGTTG---- | 42 | 67 | 109 |
| Rank-9 | -TGGAGTGTGACAATGGTGTTTT--- | 125 | 328 | 453 |
| Rank-4 | -TGGAGTGTGACAATGGTGTTT---- | 5 883 | 8 076 | 13 959 |
| Rank-8 | -TGGAGTGTGACAATGGTGTTTGCC- | 217 | 320 | 537 |
| Rank-31 | -TGGAGTGTGACAATGGTGTTTGC-- | 44 | 78 | 122 |
