# Supplementary material for: Sequencing and Bioinformatics-Based Analyses of the microRNA Transcriptome in Hepatitis B–Related Hepatocellular Carcinoma
Source: PLoS One. 2011 Jan 25;6(1):e15304. doi: 10.1371/journal.pone.0015304 (PMC3026781; doi:10.1371/journal.pone.0015304)
Supplement: Table S8 — Results of the SAM and Student's t-test. (DOC) [file pone.0015304.s010.doc]

**Supplementary Table S8.** Results of the SAM and Student’s *t*-test.

| All Genes List included into analysis |  |  |  | |  |  |  |  |  |  |  |  |
| --- | --- | --- | --- | --- | --- | --- | --- | --- | --- | --- | --- | --- |
|  |  |  |  | |  |  |  |  |  |  |  |  |
| SAM Input Parameters | | | | | |  |  |  |  |  |  |  |
| Test statistic | standard | | | | |  |  |  |  |  |  |  |
| Data Type | Two class paired | | | | |  |  |  |  |  |  |  |
| Data in log scale? | FALSE | | | | |  |  |  |  |  |  |  |
| Array centered? | FALSE | | | | |  |  |  |  |  |  |  |
| Number of Permutations | 100 | | | | |  |  |  |  |  |  |  |
| Input percentile for exchangeability factor s0 | Automatic choice | | | | |  |  |  |  |  |  |  |
| Delta | 0.78 | | | | |  |  |  |  |  |  |  |
| Number of neighbors for KNN | 10 | | | | |  |  |  |  |  |  |  |
|  |  |  |  | |  |  |  |  |  |  |  |  |
| SAM Computed Quantities | | | | | |  |  |  |  |  |  |  |
| Computed Exchangeability Factor S0 | 0.19 | | | | |  |  |  |  |  |  |  |
| S0 percentile | 0.05 | | | | |  |  |  |  |  |  |  |
| False Significant Number (Median, 90 percentile) | 0, 0 | | | | |  |  |  |  |  |  |  |
| False Discovery Rate (Median, 90 percentile) | 0, 0 | | | | |  |  |  |  |  |  |  |
| Pi0Hat | 0.69 | | | | |  |  |  |  |  |  |  |
|  |  |  |  | |  |  |  |  |  |  |  |  |
|  |  |  |  | |  |  |  |  |  |  |  |  |
| Positive Genes |  |  |  | |  |  |  |  |  |  |  |  |
| miRNA Gene Name | SAM | | | | | Paired-Student-*t* | | | | | | |
|  |  |  |  |  |  | HCC | | | ANL | | | P |
|  | Score (d) | Numerator (r) | Denominator　(s+s0) | q-value (%) | | Mean | SD | SE | Mean | SD | SE |  |
| hsa-miR-34a | 3.62 | 4.48 | 1.24 | 0.000 | | 13.39 | 7.69 | 1.81 | 8.91 | 5.65 | 1.33 | 0.000 |
| hsa-miR-21 | 3.20 | 82.77 | 25.89 | 0.000 | | 173.25 | 104.24 | 24.57 | 90.48 | 41.50 | 9.78 | 0.005 |
| hsa-miR-200c | 2.28 | 1.84 | 0.81 | 5.714 | | 4.43 | 3.43 | 0.81 | 2.59 | 2.29 | 0.54 | 0.008 |
| hsa-miR-15b | 2.12 | 2.62 | 1.23 | 5.714 | | 9.63 | 5.71 | 1.34 | 7.01 | 5.51 | 1.30 | 0.022 |
| hsa-miR-517a | 2.06 | 2.92 | 1.42 | 5.714 | | 4.97 | 5.54 | 1.31 | 2.04 | 2.53 | 0.60 | 0.029 |
| hsa-miR-19b | 1.95 | 1.58 | 0.81 | 5.714 | | 2.74 | 2.39 | 0.56 | 1.16 | 1.07 | 0.25 | 0.958 |
| hsa-miR-23a | 1.92 | 1.80 | 0.94 | 5.714 | | 3.31 | 2.51 | 0.59 | 1.51 | 1.79 | 0.42 | 0.027 |
| hsa-miR-221 | 1.89 | 6.99 | 3.71 | 5.714 | | 14.06 | 14.51 | 3.42 | 7.07 | 7.81 | 1.84 | 0.381 |
| hsa-miR-24 | 1.77 | 1.23 | 0.69 | 5.714 | | 2.56 | 2.22 | 0.52 | 1.33 | 1.48 | 0.35 | 0.025 |
| hsa-miR-27a | 1.67 | 1.47 | 0.88 | 5.714 | | 4.20 | 2.70 | 0.64 | 2.73 | 2.30 | 0.54 | 0.171 |
| hsa-miR-194 | 1.56 | 2.83 | 1.81 | 12.101 | | 11.84 | 9.84 | 2.32 | 9.01 | 7.17 | 1.69 | 0.099 |
| hsa-miR-17 | 1.54 | 0.99 | 0.64 | 12.101 | | 1.69 | 1.78 | 0.42 | 0.70 | 0.73 | 0.17 | 0.934 |
| hsa-miR-30c | 1.48 | 1.83 | 1.24 | 12.101 | | 5.70 | 3.76 | 0.89 | 3.87 | 3.40 | 0.80 | 0.096 |
| hsa-miR-25 | 1.45 | 1.69 | 1.16 | 12.101 | | 4.81 | 3.94 | 0.93 | 3.12 | 2.71 | 0.64 | 0.099 |
| hsa-let-7i | 1.42 | 1.41 | 1.00 | 12.101 | | 4.17 | 3.95 | 0.93 | 2.76 | 2.35 | 0.55 | 0.095 |
| hsa-miR-518b | 1.30 | 1.50 | 1.16 | 12.101 | | 3.44 | 4.54 | 1.07 | 1.94 | 2.14 | 0.51 | 0.137 |
| hsa-miR-100 | 1.26 | 0.94 | 0.75 | 12.101 | | 2.46 | 2.22 | 0.52 | 1.52 | 1.37 | 0.32 | 0.387 |
| hsa-miR-143 | 1.23 | 2.45 | 1.99 | 12.101 | | 10.00 | 7.08 | 1.67 | 7.55 | 5.97 | 1.41 | 0.189 |
| hsa-miR-191 | 1.23 | 2.08 | 1.69 | 12.101 | | 6.74 | 6.50 | 1.53 | 4.66 | 4.11 | 0.97 | 0.182 |
| hsa-let-7b | 1.22 | 5.89 | 4.82 | 12.101 | | 35.09 | 25.26 | 5.95 | 29.20 | 14.04 | 3.31 | 0.220 |
| hsa-miR-574-3p | 1.20 | 3.03 | 2.53 | 12.101 | | 20.07 | 19.64 | 4.63 | 17.04 | 14.75 | 3.48 | 0.212 |
| hsa-miR-497 | 1.20 | 0.99 | 0.82 | 12.101 | | 4.98 | 4.13 | 0.97 | 3.99 | 3.56 | 0.84 | 0.135 |
| hsa-miR-29b | 1.06 | 1.50 | 1.41 | 12.101 | | 3.72 | 4.78 | 1.13 | 2.22 | 2.58 | 0.61 | 0.234 |
| hsa-miR-30b | 1.06 | 1.76 | 1.66 | 12.101 | | 7.86 | 4.32 | 1.02 | 6.10 | 4.67 | 1.10 | 0.245 |
| hsa-let-7a | 1.05 | 3.32 | 3.18 | 12.101 | | 11.29 | 13.87 | 3.27 | 7.96 | 6.08 | 1.43 | 0.280 |
| hsa-miR-20a | 1.04 | 1.01 | 0.97 | 12.101 | | 1.79 | 3.37 | 0.80 | 0.78 | 0.71 | 0.17 | 0.369 |
| hsa-miR-181a | 1.04 | 1.15 | 1.11 | 12.101 | | 2.14 | 4.11 | 0.97 | 0.99 | 1.63 | 0.38 | 0.226 |
| hsa-miR-99b | 1.01 | 1.92 | 1.91 | 12.101 | | 14.23 | 14.99 | 3.53 | 12.31 | 13.72 | 3.23 | 0.276 |
| hsa-miR-7-1* | 1.01 | 1.18 | 1.17 | 12.101 | | 2.78 | 4.12 | 0.97 | 1.60 | 2.73 | 0.64 | 0.244 |
| hsa-miR-222 | 0.97 | 1.77 | 1.82 | 31.278 | | 3.56 | 6.64 | 1.56 | 1.79 | 1.98 | 0.47 | 0.292 |
| hsa-miR-26b | 0.96 | 0.49 | 0.51 | 31.278 | | 1.67 | 1.80 | 0.42 | 1.18 | 1.47 | 0.35 | 0.137 |
| MIS0018 | 0.95 | 0.61 | 0.65 | 31.278 | | 1.22 | 1.83 | 0.43 | 0.61 | 1.14 | 0.27 | 0.193 |
| hsa-miR-125a-5p | 0.90 | 0.58 | 0.65 | 31.278 | | 2.19 | 1.69 | 0.40 | 1.61 | 1.34 | 0.31 | 0.422 |
| hsa-miR-93 | 0.90 | 1.05 | 1.17 | 31.278 | | 4.11 | 3.62 | 0.85 | 3.06 | 2.85 | 0.67 | 0.297 |
| hsa-miR-23b | 0.89 | 0.84 | 0.94 | 31.278 | | 2.36 | 3.09 | 0.73 | 1.52 | 1.32 | 0.31 | 0.275 |
| hsa-miR-31 | 0.89 | 0.57 | 0.64 | 31.278 | | 1.75 | 2.37 | 0.56 | 1.18 | 2.05 | 0.48 | 0.217 |
| hsa-miR-15a | 0.87 | 0.55 | 0.64 | 31.278 | | 2.22 | 1.94 | 0.46 | 1.66 | 1.85 | 0.44 | 0.227 |
| hsa-miR-660 | 0.81 | 0.39 | 0.48 | 31.278 | | 0.54 | 1.05 | 0.25 | 0.16 | 0.36 | 0.09 | 0.186 |
| hsa-miR-124 | 0.80 | 0.53 | 0.66 | 31.278 | | 0.75 | 1.87 | 0.44 | 0.21 | 0.51 | 0.12 | 0.708 |
| hsa-miR-210 | 0.80 | 0.58 | 0.73 | 31.278 | | 0.90 | 2.14 | 0.50 | 0.32 | 0.58 | 0.14 | 0.291 |
| hsa-miR-126* | 0.75 | 0.64 | 0.86 | 31.278 | | 1.57 | 2.53 | 0.60 | 0.93 | 1.62 | 0.38 | 0.346 |
| hsa-miR-192 | 0.74 | 5.73 | 7.70 | 31.278 | | 46.10 | 31.92 | 7.52 | 40.37 | 24.85 | 5.86 | 0.456 |
| hsa-miR-26a | 0.74 | 0.79 | 1.07 | 31.278 | | 4.01 | 4.43 | 1.05 | 3.22 | 2.55 | 0.60 | 0.378 |
| hsa-miR-449a | 0.71 | 0.42 | 0.59 | 31.278 | | 1.55 | 1.67 | 0.39 | 1.13 | 1.24 | 0.29 | 0.305 |
| hsa-miR-517c | 0.67 | 0.27 | 0.40 | 31.278 | | 0.55 | 0.82 | 0.19 | 0.28 | 0.42 | 0.10 | 0.203 |
| hsa-miR-425 | 0.67 | 0.23 | 0.35 | 31.278 | | 0.35 | 0.54 | 0.13 | 0.12 | 0.36 | 0.08 | 0.146 |
| hsa-miR-199a-5p//hsa-miR-199b-5p | 0.67 | 0.39 | 0.59 | 31.278 | | 0.79 | 1.58 | 0.37 | 0.40 | 0.75 | 0.18 | 0.334 |
| hsa-let-7d | 0.65 | 0.33 | 0.50 | 31.278 | | 0.90 | 1.67 | 0.39 | 0.57 | 0.85 | 0.20 | 0.300 |
| hsa-miR-16 | 0.62 | 0.93 | 1.50 | 31.278 | | 7.29 | 4.26 | 1.00 | 6.36 | 4.24 | 1.00 | 0.487 |
| hsa-let-7a//hsa-let-7c | 0.61 | 0.37 | 0.60 | 31.278 | | 0.73 | 1.90 | 0.45 | 0.36 | 0.55 | 0.13 | 0.383 |
| hsa-let-7f | 0.60 | 0.84 | 1.39 | 31.278 | | 3.71 | 4.86 | 1.14 | 2.87 | 2.80 | 0.66 | 0.494 |
| hsa-miR-196b | 0.59 | 0.28 | 0.47 | 31.278 | | 0.49 | 0.90 | 0.21 | 0.21 | 0.66 | 0.16 | 0.327 |
| hsa-miR-214 | 0.57 | 0.62 | 1.09 | 31.278 | | 2.80 | 3.58 | 0.84 | 2.18 | 2.30 | 0.54 | 0.496 |
| hsa-miR-517a//hsa-miR-517b | 0.56 | 0.37 | 0.67 | 31.278 | | 1.24 | 1.90 | 0.45 | 0.86 | 0.99 | 0.23 | 0.437 |
| hsa-miR-512-3p | 0.54 | 0.20 | 0.37 | 31.278 | | 0.39 | 0.72 | 0.17 | 0.19 | 0.45 | 0.11 | 0.273 |
| hsa-miR-532-3p | 0.54 | 0.16 | 0.30 | 31.278 | | 0.30 | 0.45 | 0.11 | 0.14 | 0.33 | 0.08 | 0.142 |
| hsa-miR-92a | 0.54 | 0.47 | 0.88 | 31.278 | | 3.41 | 3.74 | 0.88 | 2.94 | 3.12 | 0.74 | 0.500 |
| hsa-miR-21* | 0.53 | 0.26 | 0.49 | 31.278 | | 0.66 | 1.09 | 0.26 | 0.40 | 0.60 | 0.14 | 0.386 |
| hsa-miR-320 | 0.50 | 0.28 | 0.56 | 31.278 | | 0.85 | 1.52 | 0.36 | 0.57 | 0.85 | 0.20 | 0.452 |
| hsa-miR-451 | 0.47 | 0.54 | 1.16 | 31.278 | | 1.71 | 3.99 | 0.94 | 1.16 | 1.10 | 0.26 | 0.578 |
| hsa-miR-200a | 0.43 | 0.22 | 0.51 | 31.278 | | 0.67 | 0.99 | 0.23 | 0.45 | 0.78 | 0.18 | 0.494 |
| hsa-miR-98 | 0.41 | 0.18 | 0.42 | 31.278 | | 0.54 | 0.94 | 0.22 | 0.36 | 0.68 | 0.16 | 0.452 |
| hsa-miR-503 modified | 0.38 | 0.18 | 0.48 | 31.278 | | 1.98 | 2.38 | 0.56 | 1.79 | 1.91 | 0.45 | 0.535 |
| hsa-miR-196a | 0.32 | 0.15 | 0.46 | 31.278 | | 0.49 | 0.72 | 0.17 | 0.34 | 0.87 | 0.21 | 0.587 |
| hsa-miR-200b | 0.32 | 0.30 | 0.94 | 31.278 | | 2.68 | 2.77 | 0.65 | 2.38 | 2.63 | 0.62 | 0.695 |
| hsa-miR-103 | 0.29 | 0.13 | 0.45 | 31.278 | | 0.54 | 0.75 | 0.18 | 0.41 | 0.58 | 0.14 | 0.620 |
| hsa-miR-29a | 0.25 | 0.23 | 0.91 | 31.278 | | 1.89 | 2.33 | 0.55 | 1.66 | 2.42 | 0.57 | 0.755 |
| hsa-miR-524-5p | 0.24 | 0.10 | 0.41 | 31.278 | | 0.41 | 0.78 | 0.18 | 0.31 | 0.47 | 0.11 | 0.651 |
| hsa-miR-527//518a-5p | 0.23 | 0.08 | 0.34 | 31.278 | | 0.22 | 0.44 | 0.10 | 0.14 | 0.45 | 0.11 | 0.596 |
| hsa-let-7e | 0.21 | 0.12 | 0.58 | 31.278 | | 0.80 | 1.34 | 0.32 | 0.68 | 0.97 | 0.23 | 0.189 |
| hsa-miR-125b | 0.21 | 0.98 | 4.68 | 31.278 | | 30.83 | 17.12 | 4.03 | 29.85 | 15.98 | 3.77 | 0.830 |
| hsa-miR-151-5p | 0.13 | 0.08 | 0.59 | 31.278 | | 1.39 | 1.58 | 0.37 | 1.31 | 1.55 | 0.36 | 0.843 |
| hsa-miR-126 | 0.13 | 0.08 | 0.64 | 31.278 | | 2.14 | 2.75 | 0.65 | 2.05 | 2.07 | 0.49 | 0.851 |
| hsa-miR-103//hsa-miR-107 | 0.12 | 0.06 | 0.49 | 31.278 | | 0.66 | 0.81 | 0.19 | 0.60 | 0.83 | 0.19 | 0.839 |
| hsa-miR-27b | 0.12 | 0.15 | 1.18 | 31.278 | | 4.52 | 3.03 | 0.71 | 4.38 | 3.88 | 0.91 | 0.884 |
| hsa-miR-130a | 0.09 | 0.07 | 0.81 | 31.278 | | 1.67 | 2.03 | 0.48 | 1.60 | 1.89 | 0.45 | 0.910 |
| hsa-miR-106b* | 0.04 | 0.02 | 0.45 | 31.278 | | 0.62 | 0.70 | 0.16 | 0.60 | 0.84 | 0.20 | 0.942 |
| hsa-miR-202* | 0.01 | 0.01 | 0.76 | 31.278 | | 1.10 | 1.71 | 0.40 | 1.09 | 1.56 | 0.37 | 0.987 |
|  |  |  |  |  | |  |  |  |  |  |  |  |
| Negative Genes |  |  |  |  | |  |  |  |  |  |  |  |
| miRNA Gene Name | SAM | | | | | Paired-Student-t | | | | | | |
|  |  |  |  |  |  | HCC | | | ANL | | | P |
|  | Score (d) | Numerator (r) | Denominator　(s+s0) | q-value (%) | | Mean | SD | SE | Mean | SD | SE |  |
| hsa-miR-122 | -3.92 | -168.83 | 43.10 | 0.000 | | 168.95 | 111.32 | 26.24 | 337.78 | 173.33 | 40.85 | 0.001 |
| hsa-miR-122* | -2.25 | -3.14 | 1.39 | 6.234 | | 2.56 | 7.29 | 1.72 | 5.70 | 10.46 | 2.47 | 0.018 |
| hsa-miR-138 | -1.36 | -0.74 | 0.54 | 6.234 | | 0.46 | 1.18 | 0.28 | 1.20 | 2.18 | 0.51 | 0.049 |
| hsa-miR-146a | -1.23 | -0.74 | 0.60 | 6.234 | | 0.36 | 0.55 | 0.13 | 1.09 | 1.56 | 0.37 | 0.086 |
| hsa-miR-195 | -1.16 | -0.60 | 0.52 | 6.234 | | 0.68 | 1.02 | 0.24 | 1.28 | 1.24 | 0.29 | 0.082 |
| hsa-miR-503 non-modified | -0.99 | -0.32 | 0.32 | 6.234 | | 0.09 | 0.26 | 0.06 | 0.41 | 0.58 | 0.14 | 0.025 |
| hsa-miR-455-3p | -0.91 | -0.63 | 0.69 | 6.234 | | 0.78 | 1.51 | 0.36 | 1.41 | 2.61 | 0.61 | 0.221 |
| hsa-miR-342-3p | -0.91 | -0.37 | 0.41 | 6.234 | | 0.34 | 0.79 | 0.19 | 0.71 | 1.19 | 0.28 | 0.101 |
| hsa-let-7g | -0.79 | -0.44 | 0.56 | 6.234 | | 1.49 | 2.67 | 0.63 | 1.93 | 2.45 | 0.58 | 0.245 |
| hsa-miR-142-3p | -0.69 | -0.77 | 1.11 | 6.234 | | 3.29 | 2.36 | 0.56 | 4.06 | 4.13 | 0.97 | 0.414 |
| hsa-miR-140-3p | -0.67 | -0.65 | 0.98 | 6.234 | | 3.27 | 3.45 | 0.81 | 3.92 | 3.00 | 0.71 | 0.414 |
| hsa-miR-99a | -0.66 | -0.88 | 1.33 | 6.234 | | 4.06 | 2.88 | 0.68 | 4.93 | 4.82 | 1.14 | 0.449 |
| hsa-miR-29c | -0.59 | -0.23 | 0.39 | 6.234 | | 0.21 | 0.43 | 0.10 | 0.44 | 0.66 | 0.15 | 0.252 |
| hsa-miR-424 | -0.57 | -0.59 | 1.03 | 6.234 | | 4.65 | 4.39 | 1.03 | 5.24 | 3.29 | 0.78 | 0.007 |
| hsa-miR-199a-5p | -0.51 | -0.41 | 0.79 | 6.234 | | 1.72 | 2.41 | 0.57 | 2.13 | 2.18 | 0.51 | 0.504 |
| hsa-miR-199a-3p//199b-3p | -0.50 | -0.89 | 1.79 | 6.234 | | 7.97 | 4.81 | 1.13 | 8.87 | 5.67 | 1.34 | 0.583 |
| hsa-miR-486-5p | -0.41 | -0.30 | 0.74 | 6.234 | | 0.99 | 1.15 | 0.27 | 1.29 | 1.72 | 0.40 | 0.591 |
| hsa-miR-525-5p | -0.40 | -0.19 | 0.48 | 6.234 | | 0.52 | 1.11 | 0.26 | 0.71 | 0.97 | 0.23 | 0.516 |
| has-miR-672 | -0.38 | -0.31 | 0.82 | 6.234 | | 1.07 | 1.71 | 0.40 | 1.37 | 1.71 | 0.40 | 0.628 |
| hsa-miR-145 | -0.38 | -0.22 | 0.59 | 6.234 | | 1.29 | 2.53 | 0.60 | 1.51 | 1.50 | 0.35 | 0.582 |
| hsa-miR-141 | -0.21 | -0.09 | 0.46 | 6.234 | | 0.57 | 0.81 | 0.19 | 0.67 | 0.99 | 0.23 | 0.724 |
| hsa-let-7c | -0.19 | -0.38 | 2.07 | 6.234 | | 16.54 | 8.47 | 2.00 | 16.93 | 7.38 | 1.74 | 0.840 |
| hsa-miR-885-5p | -0.15 | -0.11 | 0.74 | 6.234 | | 0.78 | 1.93 | 0.45 | 0.89 | 1.68 | 0.40 | 0.842 |
| hsa-miR-30d | -0.12 | -0.13 | 1.12 | 6.234 | | 3.16 | 2.39 | 0.56 | 3.29 | 2.93 | 0.69 | 0.776 |
| hsa-miR-30a | -0.10 | -0.05 | 0.50 | 6.234 | | 0.61 | 1.03 | 0.24 | 0.67 | 0.73 | 0.17 | 0.865 |
| hsa-miR-519a*//519c-5p//519b-5p//523*//518e*//522 | -0.02 | -0.01 | 0.67 | 6.234 | | 0.67 | 1.50 | 0.35 | 0.69 | 1.33 | 0.31 | 0.981 |
| hsa-miR-519a | -0.02 | -0.01 | 0.67 | 6.234 | | 0.67 | 1.50 | 0.35 | 0.69 | 1.33 | 0.31 | 0.981 |
