# Supplementary material for: Sequencing and Bioinformatics-Based Analyses of the microRNA Transcriptome in Hepatitis B–Related Hepatocellular Carcinoma
Source: PLoS One. 2011 Jan 25;6(1):e15304. doi: 10.1371/journal.pone.0015304 (PMC3026781; doi:10.1371/journal.pone.0015304)
Supplement: Table S9 — Discriminating HCC from adjacent normal liver using miRNA. The true-positives (sensitivity) and false-positives (1-specificity), together with the positive predictive values, negative predictive values, likelihood ratio for positive predictive value, negative predictive value, likelihood ratio for positive predictive value, and accuracy at various cut-off points are listed. (DOC) [file pone.0015304.s011.doc]

**Supplementary Table S9. Discriminating HCC from adjacent normal liver using miRNA. The true-positives (sensitivity) and false-positives (1-specificity), together with the positive predictive values, negative predictive values, likelihood ratio for positive predictive value, negative predictive value, likelihood ratio for positive predictive value, and accuracy at various cut-off points are listed.**

| Cut  off  point  (No. of Clones) | Sensitivity | Specificity | Positive  predictive  value | Negative  predictive  value | Likelihood  ratio for  positive  results | Likelihood  ratio for  negative  results | Accuracy |
| --- | --- | --- | --- | --- | --- | --- | --- |
| miR-21 | | | | | | | |
| 90 | 89 | 44 | 62 | 80 | 1.6 | 0.25 | 67 |
| 100 | 78 | 72 | 74 | 76 | 2.8 | 0.31 | 75 |
| 110 | 72 | 72 | 72 | 72 | 2.6 | 0.38 | 72 |
| 120 | 61 | 83 | 79 | 68 | 3.67 | 0.47 | 72 |
| miR-122 | | | | | | | |
| 150 | 44 | 78 | 67 | 58 | 2.0 | 0.71 | 61 |
| 200 | 72 | 72 | 72 | 72 | 2.6 | 0.38 | 72 |
| 250 | 83 | 72 | 75 | 81 | 3.0 | 0.23 | 78 |
| 300 | 89 | 56 | 67 | 83 | 2.0 | 0.20 | 72 |
