# Supplementary material for: Sequencing and Bioinformatics-Based Analyses of the microRNA Transcriptome in Hepatitis B–Related Hepatocellular Carcinoma
Source: PLoS One. 2011 Jan 25;6(1):e15304. doi: 10.1371/journal.pone.0015304 (PMC3026781; doi:10.1371/journal.pone.0015304)
Supplement: Table S10 — Analysis of the association between the postoperative clinical course and variables. The postoperative recurrence and survival of patients with HCC in relation to pathologic tumor characteristics and miRNA expression calculated by clone counts are analyzed. (DOC) [file pone.0015304.s012.doc]

**Supplementary Table S10.** Analysis of the association between the postoperative clinical course and variables. The postoperative recurrence and survival of patients with HCC in relation to pathologic tumor characteristics and miRNA expression calculated by clone counts are analyzed.

| Variables | Subset | Recurrence | | Survival | |
| --- | --- | --- | --- | --- | --- |
|  |  | Hazard ratio (95% Confidence interval) | *P* | Hazard ratio (95% Confidence interval) | *P* |
| Age^†^ |  | 1.00 (0.95-1.06) | 1.000 | 1.00 (0.94-1.07) | 0.949 |
| Gender | male/female | 24.09 (0-230388) | 0.496 | 23.07 (0-2991627) | 0.601 |
| Stage (TNM) | IV/I-III | 3.61 (0.81-16.16) | 0.093 | 10.47 (1.25-87.93) | 0.030 |
| Tumor size^†^ |  | 1.08 (0.93-1.24) | 0.283 | 1.09 (0.97-1.23) | 0.146 |
| Differentiation | poor/well・moderately | 1.66 (0.34-8.09) | 0.508 | 0.39 (0.18-12.26) | 0.388 |
| Growth type | ig/eg | 2.08 (0.52-8.36) | 0.303 | 1.50 (0.29-7.77) | 0.627 |
| capsule formation | +/- | 1.70 (0.33-8.89) | 0.528 | 6.23 (1.37-28.32) | 0.018 |
| capsule invasion | +/- | 1.15 (0.23-5.77) | 0.864 | 4.28 (0.84-21.73) | 0.079 |
| serosa invasion | +/- | 3.20 (0.62-16.57) | 0.165 | 6.96 (1.54-31.46) | 0.012 |
| septum formation | +/- | 4.67 (0.89-204.34) | 0.068 | 1.31 (0.16-10.95) | 0.803 |
| portal vein invasion | +/- | 2.35 (0.47-11.70) | 0.296 | 36.66 (0.43-31168) | 0.295 |
| venous invasion | +/- | 3.81 (0.76-19.06) | 0.104 | 4.06 (0.49-33.77) | 0.195 |
| arterial invasion | +/- | 3.59 (0.76-16.97) | 0.106 | 2.72 (0.61-12.23) | 0.192 |
| bile duct invasion | +/- | 2.77 (0.50-15.26) | 0.241 | 2.66 (0.50-14.11) | 0.250 |
| intrahepatic metastasis | +/- | 5.43 (0.98-30.14) | 0.053 | 5.09 (0.91-28.44) | 0.064 |
| Cluster 2 | yes/no | 5.44 (1.42-20.80) | 0.013 | 3.49 (0.76-15.67) | 0.103 |

^*^Results from the Cox proportional hazards regression analysis.

^†^Continuous variable.
