# Supplementary material for: Sequencing and Bioinformatics-Based Analyses of the microRNA Transcriptome in Hepatitis B–Related Hepatocellular Carcinoma
Source: PLoS One. 2011 Jan 25;6(1):e15304. doi: 10.1371/journal.pone.0015304 (PMC3026781; doi:10.1371/journal.pone.0015304)
Supplement: Table S11 — Cox regression analysis of the association between the postoperative clinical course and variables. (DOC) [file pone.0015304.s013.doc]

**Supplementary Table S11** Cox regression analysis of the association between the postoperative clinical course and variables.

| Recurrence |  |  |  |
| --- | --- | --- | --- |
| Variables | Subset | Hazard ratio (95%Confidence interval) | *P* |
| Cluster 2 | yes/no | 5.27 (1.28-21.78) | 0.022 |
| intrahepatic metastasis | +/- | 6.35 (0.99-40.76) | 0.051 |
|  |  |  | |
| Survival |  |  | |
| Variables | Subset | Hazard ratio (95%Confidence interval) | *P* |
| capsule formation | +/- | 19.60 (1.73-222.27) | 0.016 |

^*^ Results obtained with the use of a forward, stepwise multiple Cox proportional hazards regression model.
